# A dense SNP-based linkage map for Atlantic salmon (*Salmo salar*) reveals extended chromosome homeologies and striking differences in sex-specific recombination patterns

**DOI:** 10.1186/1471-2164-12-615

**Published:** 2011-12-19

**Authors:** Sigbjørn Lien, Lars Gidskehaug, Thomas Moen, Ben J Hayes, Paul R Berg, William S Davidson, Stig W Omholt, Matthew P Kent

**Affiliations:** 1 Centre for Integrative Genetics (CIGENE) and Department of Animal and Aquacultural Sciences, Norwegian University of Life Sciences, N-1432, Ås, Norway; 2 Aqua Gen AS, Postboks 1240, Pirsenteret, 7462 Trondheim, Norway; 3 Biosciences Research Division, Department of Primary Industries Victoria, 1 Park Drive, Bundoora 3083, Australia; 4 Department of Molecular Biology and Biochemistry, Simon Fraser University, Burnaby BC, V5A 1S6, Canada

## Abstract

**Background:**

The Atlantic salmon genome is in the process of returning to a diploid state after undergoing a whole genome duplication (WGD) event between 25 and100 million years ago. Existing data on the proportion of paralogous sequence variants (PSVs), multisite variants (MSVs) and other types of complex sequence variation suggest that the rediplodization phase is far from over. The aims of this study were to construct a high density linkage map for Atlantic salmon, to characterize the extent of rediploidization and to improve our understanding of genetic differences between sexes in this species.

**Results:**

A linkage map for Atlantic salmon comprising 29 chromosomes and 5650 single nucleotide polymorphisms (SNPs) was constructed using genotyping data from 3297 fish belonging to 143 families. Of these, 2696 SNPs were generated from ESTs or other gene associated sequences. Homeologous chromosomal regions were identified through the mapping of duplicated SNPs and through the investigation of syntenic relationships between Atlantic salmon and the reference genome sequence of the threespine stickleback (*Gasterosteus aculeatus*). The sex-specific linkage maps spanned a total of 2402.3 cM in females and 1746.2 cM in males, highlighting a difference in sex specific recombination rate (1.38:1) which is much lower than previously reported in Atlantic salmon. The sexes, however, displayed striking differences in the distribution of recombination sites within linkage groups, with males showing recombination strongly localized to telomeres.

**Conclusion:**

The map presented here represents a valuable resource for addressing important questions of interest to evolution (the process of re-diploidization), aquaculture and salmonid life history biology and not least as a resource to aid the assembly of the forthcoming Atlantic salmon reference genome sequence.

## Background

Atlantic salmon (*Salmo salar*) belongs to the subfamily Salmoninae in the order Salmoniformes. The common ancestor of the salmonid fishes is suspected to have undergone a whole genome duplication (WGD) 25-100 million years ago [1]. Today, salmonids have karyotypes containing various numbers of metacentric and acrocentric chromosomes likely arising through Robertsonian fissions and fusions of ancestral acrocentric chromosomes [2]. Atlantic salmon possesses a karyotype with 72-74 chromosome arms, compared to approximately 100 chromosome arms found in other family members. The presence of multivalent pairing at meiosis and evidence of tetrasomic inheritance [3] suggest that the post-tetraploidization return to disomic inheritance is not yet complete. A model of "secondary tetrasomy" in which homologous chromosomes first pair and then recombine in regions proximal to the centromere before undergoing homeologous pairing and recombination toward the distal end of the chromosome has been suggested for the salmonid species [1]. Further, Ohno et al. [4] suggested that the genomes of salmonids have been reverting toward a diploid state through the differentiation of duplicated chromosome sets into distinct pairs of homeologs. How an inherently unstable duplicated genome reverts to a stable diploid state is poorly understood. Although large-scale deletions, gene silencing and chromosomal rearrangements are all thought to be a part of this process, it is not known if these events occur randomly along different lineages or if there is a burst of activity immediately after the duplication followed by stability in the resulting genomes [5].

A linkage map constructed from carefully chosen genetic markers could be a valuable tool to investigate some of these questions. Marker development and construction of genetic maps characterizing the inheritance patterns of traits and markers have proven invaluable for addressing important biomedical, agricultural, ecological and evolutionary questions. Genetic maps in species of the subfamily Salmoninae [6-15], including Atlantic salmon, have relied primarily on microsatellite markers. The biallelic nature of single nucleotide polymorphisms (SNPs) makes them less informative, but they are abundant and highly suitable for cost effective high-throughput genotyping. As a result SNPs have emerged as the genetic marker of choice for large-scale linkage and association studies, genomic predictions, pedigree and broodstock analysis, linkage disequilibrium and haplotype mapping, and even population biology studies. If SNPs are developed from sequences associated with genes (e.g. expressed sequence tags; ESTs) they become particularly useful in comparative mapping. The interpretation of SNP data in Atlantic salmon is complicated by the WGD, which has made it difficult to differentiate between single locus SNPs and the more complex paralogous sequence variants (PSVs) and multisite variants (MSVs), which arise from gene duplicates [16,17].

Recently we have developed an Illumina iSelect SNP genotyping chip, containing approximately 6 K SNP-assays. Approximately half of the SNPs on the array were identified from EST alignments [18,19], with most of the remainder coming from 454 sequencing of a random genomic sample collected by preparing reduced representation libraries from individual and pooled DNA samples. We used this chip to genotype a large set of family material from a Norwegian aquacultural population and to construct a dense linkage map for Atlantic salmon. The fact that half of the SNPs were developed from ESTs or other gene associated sequences, facilitated identification of homeologous regions in Atlantic salmon genome and allowed us to build syntenic relationships between Atlantic salmon and the reference genome sequence of the threespine stickleback.

## Results and discussion

### Linkage map construction

Linkage mapping based on genotyping of 3297 Atlantic salmon from 143 families resulted in a linkage map with a total of 5650 SNPs. Linkage groups were translated to chromosomes following the nomenclature suggested by Phillips et al. [20] after comparing positions of markers included in the studies of Moen et al. [21], Danzmann et al. [6] and Lorenz et al. [22]. Our linkage mapping identified 29 linkage groups which agrees with the most common karyotype in European Atlantic salmon. Thus, the 29 linkage groups reported here likely correspond to the 29 chromosome pairs of the 'European karyotype' reported by Phillips et al. [20].

### Difference in recombination patterns between sexes

Previous studies in Atlantic salmon have reported extreme differences in recombination rates between the sexes, with female:male ratios ranging from ~5:1 to 8.26:1 in two studies of farmed Norwegian salmon [8,21] and 7.05:1 to 7.23:1 in two SALMAP families [6]. In contrast to these previous reports, the linkage maps presented in this study (Table 1) show a smaller overall difference in recombination rate between sexes (1.38:1). The reason for this marked difference in reported recombination rates is likely to be our maps improved marker coverage in telomeric regions. Since male recombination is often elevated in telomeres [6,21] the more comprehensive coverage of these regions in our study has resulted in a more even recombination rate between sexes. The largest sex related differences in recombination rate were found for chromosomes ssa02, ssa08 and ssa17 showing female:male ratios ranging from 4.51:1 to 7.39:1. In contrast seven of the smaller chromosomes (ssa15, ssa19, ssa21, ssa22, ssa23, ssa25 and ssa27) showed similar or higher recombination rate in males than females (see Table 1). Thus, the results clearly show that the differences between sexes, with regards to genetic recombination, lie primarily in the distribution of recombination events, not in the total map lengths. These results are supported by the most recent linkage maps constructed for rainbow trout [13] and it is quite likely that the male and female map lengths will converge further when marker density is increased.

**Table 1:** Summary of the Atlantic salmon linkage map

		Linkage group size (cM)		
			
Chromosome	Number of SNPs	Female map	Male map	Female:male ratio
ssa01	386	135.3	130.1	1.04

ssa02	241	121.8	27	4.51

ssa03	291	115.4	61	1.89

ssa04	224	112.4	99.1	1.13

ssa05	255	116.6	54.9	2.12

ssa06	251	119.9	68.4	1.75

ssa07	158	114	72.2	1.58

ssa08	71	56.2	7.6	7.39

ssa09	311	106.8	79.1	1.35

ssa10	296	88.1	65.8	1.34

ssa11	233	85.1	58.4	1.46

ssa12	242	118.6	66.5	1.78

ssa13	285	89.7	84.1	1.07

ssa14	206	69.2	65.1	1.06

ssa15	215	80.1	82.3	0.97

ssa16	192	63	18.6	3.39

ssa17	166	69.3	11.3	6.13

ssa18	164	73.8	37.7	1.96

ssa19	157	66.3	71.6	0.93

ssa20	177	63.3	49.2	1.29

ssa21	107	53.9	66.7	0.81

ssa22	160	58.8	62.5	0.94

ssa23	126	51.4	58.1	0.88

ssa24	115	58.7	58.6	1.00

ssa25	117	55	56.2	0.98

ssa26	145	81.6	77.5	1.05

ssa27	162	53.6	57.7	0.93

ssa28	96	53.7	44.9	1.20

ssa29	101	71.4	60.7	1.18

	**5650**	**2403**	**1752.9**	**1.37**

**Figure 1:**
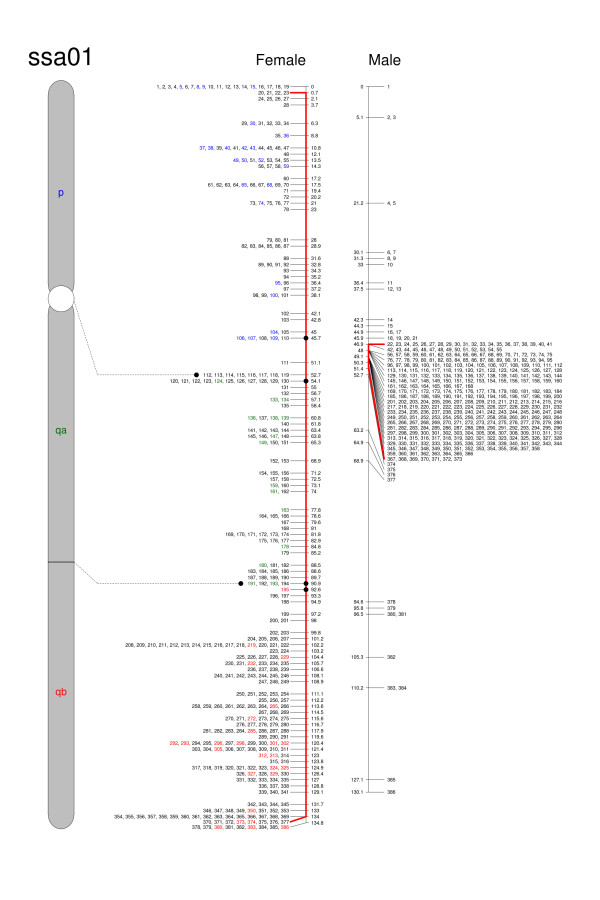
**Sex specific linkage maps for Atlantic salmon chromosome 1 (ssa01)**. The physical size and appearance of the chromosome is adopted from Phillips et al. (2009). Approximate genetic distances are given to the right of the female map and to the left of the male map. The numbers opposite denote indexes to individual markers as given in Additional Data. Markers homologous to chromosome arm segments in stickleback or salmon are indicated with colours correponding with one of the chromosome arms. The approximate separation of the female genetic map is based on this information. Red lines extend to the set of markers with limited recombination in the male map. Large regional differences are seen between the maps, with most of the male recombination taking place near the telomeric ends.

Maps for the 29 chromosomes including marker information, informative meioses and map positions are presented in Additional file 1 (SalmonLinkageMapSept2010.txt). Previous studies in Atlantic salmon have also reported large regional differences in recombination between the two sexes where females recombine all along the chromosomes and males show strongly localized recombination in telomeric regions [6]. Increased recombination in telomeric regions for some male chromosomes was evident in our study. This is exemplified in Figure 1, which is an illustration of chromosome ssa01 displaying both the male and female maps. For seven metacentric chromosomes (ssa02 to ssa08), however, we detected elevated recombination at only one of the telomeres. The regional recombination pattern differences between sexes are illustrated in linkage maps presented in Additional file 2 (chromosomes.zip).

### Clustering and mapping of MSVs

**Figure 2:**
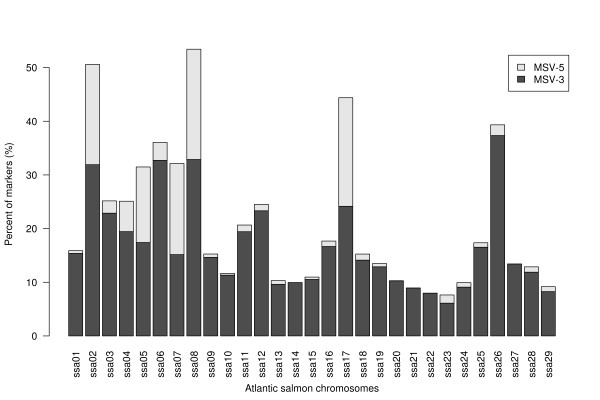
**The proportion of MSVs as a percentage of the total number of markers for each Atlantic salmon chromosome**.

Striking evidence for the salmonid WGD event is present today in the Atlantic salmon genome. SNP markers within duplicated sequences (multisite-variants; MSVs [23]) differ from typical single locus diploid SNPs in that their assay signal reflects a mixture of four alleles (two alleles in each duplicon). MSVs are further subdivided into MSV-3s; where only one duplicon or paralogue is variable, and MSV-5s; where both paralogues are variable. The nomenclature is chosen to reflect three or five cluster patterns observed when data is inspected using Illumina's GenomeStudio Genotyping Analysis Module (Illumina, San Diego, CA). Based on automated clustering of genotype data using the beadarrayMSV [17] and manual inspection of data using the GenomeStudio Genotyping Analysis Module (Illumina, San Diego, CA) we estimate that approximately 21% of markers are MSVs, which is higher than the 9.5% previously estimated from microsatellite data [6]. Despite the random nature of SNP discovery, the MSVs do not distribute evenly across all linkage groups (see Figure 2). For some chromosomes, > 40% of mapped markers are MSVs (ssa02, ssa08, ssa17 and ssa26), while in contrast many other chromosomes contain fewer than 10% MSVs (ssa13, ssa14, ssa15, ssa20, ssa21, ssa22, ssa23, ssa24 and ssa25), reflecting the pseudo-tetraploidy of the salmon genome. The apparent nonrandom distribution of MSVs argues strongly for the development of tools that can be applied for automated scoring (clustering), filtering and mapping of complex SNPs, as discarding such markers will bias map construction and create information gaps in the linkage map. Due to the regional differences in recombination rates between sexes, such information gaps may also be sex-biased. Recently an R-package, beadarrayMSV, has been developed for analysis of SNP markers in mosaic tetraploid species [17]. The program package also enables efficient scoring and integration of markers that are variable in both paralogs (MSV-5s).

### Identification of homeologous chromosome regions

It has been suggested that the common ancestor of the salmonids had a typical teleost karyotype comprising 24-25 pairs of acrocentric chromosomes [24]. If true, then it would be reasonable to expect 24-25 pairs of duplicated regions in the present-day salmonids. A recent study investigating conservation of large syntenic blocks between Atlantic salmon and rainbow trout supports this hypothesis by revealing good correspondence between 50 haploid chromosome arms in rainbow trout and equivalent segments in Atlantic salmon [20]. We have investigated the hypothesis by assessing the positions of MSV-5s on the linkage map. As shown in Table 2 the majority of MSV-5s map to chromosomes 2, 3, 4, 5, 6, 7 and 17. More specifically, mapping of MSVs segregating at both of their homeologous loci suggests homeologies between 2p-5q, 3q-6p, 4p-8q and 7q-17qb. A smaller number of MSV-5s suggest homeology between 2q-12qa, 11qa-26, 13qa-15qb and 19qb-29.

**Table 2:** Homeologous chromosome regions in the Atlantic salmon genome identified by mapping of MSV-5s, sequence alignments within salmon SNP-sequences (Salmon BLAST) and alignments against the stickleback genome sequence (Stickleback BLAST)

	Mapping approach			
			
Homeologs	MSV-5s	Salmon BLAST	Stickleback BLAST	Danzmann et al (2008)
1p-9qa		4	37	

1qa-18qa		3	29	

1qb-11qb/13qb		6	52	

2p-5q	39	48	67	9

2q-12qa	1	5	33	5 (12qb)

3p-14qa			41	

3q-6p	7	14	67	9

4p-8q	14	16	21	4

4q-11qb/13qb		11	58	

5p-9qb		7	61	

6q-15qa		3	41	

7p-18qb			18	

7q-17qb	33	34	35	5 (17qa)

9qc-20qa		4	36	

10-16qa/23		3	112	

11qa-26	2	10	54	1

12qb-22		7	56	

13qa-15qb	1	7	40	

14qb-27		11	57	

16qb-17qa		7	19	5 (16qa-17qb)

18qa-28			14	

19qa-28		4	24	

19qb-29	1	3	38	

20qb-24		5	52	

21-25		5	51	

MSV-5s are nested in the Salmon BLAST and Stickleback BLAST numbers are the total counts of SNP-bearing sequences aligning with the Stickleback genome and supporting the homeolog pairing.

To validate these putative homeologies, and to identify other homeologous regions, sequences flanking mapped SNPs and MSVs were aligned against each other using BLAST [25] to identify paralogs. The BLAST analysis generated a list of 206 SNP-bearing sequences with BLAST similarities to two Atlantic salmon chromosomes, for which 98 had been previously identified from MSV-5 data (Table 2). To safeguard against false positives homeology between two chromosomes was determined by a minimum of three SNP-bearing sequences. This search provided additional evidence for the eight homeologous regions identified by MSV-5 mapping and highlighted 14 new regions, producing a total of 22 regions (Table 2). Among these, six homeology predictions have been reported in previous studies [6,20].

**Figure 3:**
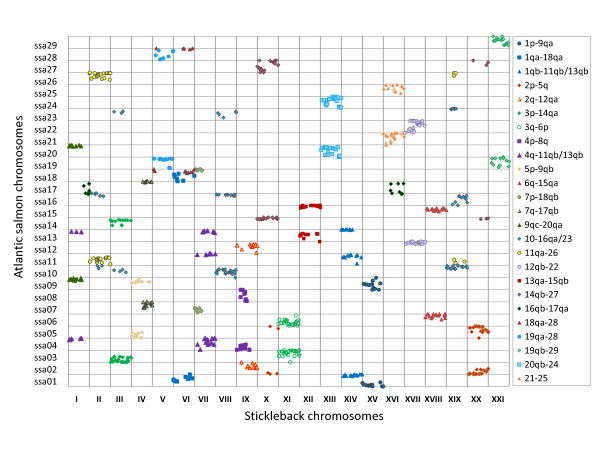
**Figure reporting BLAST matches between markers on homeologous Atlantic salmon chromosomes and stickleback chromosomes**. Patterns exist where groups of mapped SNPs from different Salmon chromosomes match a particular chromosome or region in stickleback. Within each grid square the relative hit positions are indicated horizontally for stickleback and vertically for salmon chromosomes. For example, SNPs on Ssa13qa and 15qb (red squares) align to positions across the full length of stickleback chromosome 12, providing evidence that Ssa13qa and 15qb are homeologues.

To further develop our understanding of homeology within Atlantic salmon, sequences flanking mapped SNPs and MSVs were compared with the stickleback reference genome. Our ability to identify reliable BLAST similarities in stickleback was strengthened considerably by the high proportion of salmon markers (2696) generated from ESTs or other gene associated sequences. Once again, three BLAST similarities for each chromosome were required to assert synteny between stickleback and homeologous regions in salmon. As shown in Figure 3, the alignment produced clear patterns where groups of mapped salmon SNPs matched up with particular chromosomal regions in stickleback. No discrepancies in homeologies were found when comparing the results from this approach to the other two approaches described above. The alignment against stickleback increased the number of paired homeologous regions to 25 (Table 2) which supports the results of Phillips et al. [20]. Most homeologies were unambiguous, with the exception of the homeologs of ssa1qb and ssa4q which seems to be a fusion of ssa11qb and ssa13qb, and ssa10 for which we could not uniquely separate matching regions on ssa16qa and ssa23.

### Recombination patterns and the post-tetraploidization process

A key event during re-diploidization is the switch from tetrasomic to disomic inheritance, i.e. from having four chromosomes forming a quadrivalent to having two pairs each forming a bivalent during meiosis. Most present-day salmonids seem to have restored disomic inheritance across most of the genome as a part of the post-tetraploidization events, but meiotic multivalent and tetrasomic inheritance, especially in males, is well documented for several species within the Salmonidae e.g., rainbow trout [3] and brown trout [9]. The origin of such sex differences remains unclear, although it has been suggested that multivalent formation may be constrained during female meiosis due to greater specificity in the initiation of chromosome pairing relative to males [9]. A two-stage model of chromosome pairing has been proposed in which homologous chromosomes pair first to ensure disjunction of homeologs, followed by pairing between homeologous regions [1]. According to this model, loci near the centromeres would show disomic inheritance while more distal loci show tetrasomic inheritance, or secondary tetrasomy. Homeologous chromosomal regions, which form multivalents with their ancestral counterpart during meiosis, are believed to retard re-diploidization while segments more proximal to the centromere are allowed to diverge at a faster rate [1]. Our linkage data in combination with mapping of homeologous regions in the salmon genome provide substantial support for the presence of these mechanisms in Atlantic salmon. The majority of chromosomes are characterized by strongly localized recombination towards telomere regions in males. However, for seven metacentric chromosomes (ssa02 to ssa08) elevated recombination was found only at one of the telomeres. For the majority of these chromosome arms (5q, 3q, 4p, 6p, and 7q), low recombination rates in males coincides with extensive homeologies between chromosome pairs (2p-5q, 3q-6p, 4p-8q and 7q-17qb) demonstrated by the presence of many MSV-5s. Further support for homeologous chromosome pairing in these regions is given by two-point linkage analyses, which show considerable pseudo-linkage between markers on 2p-5q, 3q-6p, 4p-8q and 7q-17qb (results not shown). Competitive crossing-over in the quadrivalents during meiosis may explain why the expected, increased male recombination is not observed in some of these regions as recombination between homeologs prevents recombination between sister chromatids. In contrast both 2p and 8q seem to have elevated recombination at the telomere ends showing homeology with other chromosome regions. These chromosome arms contain heterochromatin rich regions which generate a strong signal with DAPI staining [20]. Heterochromatin rich regions may reduce crossing over [26] which may serve as an explanation for this difference. Notably, both ssa02 and ssa08, together with ssa17 which builds extensive homeology with 7q, demonstrate the highest female:male recombination rates in our dataset. It is therefore tempting to suggest that the homeolog chromosome pairing causes an overall reduction in male recombination for these three chromosomes.

Over the last decade researchers have begun to include genetic marker information in their strategies to improve salmonid aquaculture production efficiency [27]. Although chromosomal segments have been identified that are associated with economically important traits (e.g. [28-31]) the density of available marker maps has been insufficient for performing whole genome wide association (GWA) mapping in Atlantic salmon. The development of the 6 K SNP-chip, together with the dense linkage map presented in this paper, provide significant steps forward towards fine-mapping quantitative trait loci (QTL) affecting health and productivity, as well as revealing the biological mechanisms underlying these traits.

To date, no whole genome sequence exists for any member of the family Salmonidae. Thus building syntenic relationships with the sequenced and functionally well-characterized model teleost species zebrafish (*Danio rerio*), fugu (*Fugu rubripes*), Tetraodon (*Tetraodon nigroviridis*), medaka (*Oryzias latipes*), and three-spined stickleback (*Gasterosteus aculeatus*) may extrapolate valuable information. Although the data presented broadly reveals conservation of synteny groups between Atlantic salmon and stickleback, significant rearrangements of synteny blocks are expected along comparative chromosomes. As the densities and resolution of markers in the Atlantic salmon linkage maps increase, and the sequencing and assembly of the salmon genome advance, the extent of such rearrangements will be better resolved.

Work is in progress to generate a complete reference genome for Atlantic salmon [32]. The high-resolution linkage map presented in this work can be integrated with other data and become a valuable resource to guide and assist assembly of the very complex salmon genome. Even after the salmon genome is completely sequenced, the map will continue to be a useful tool to link observable phenotypes and animal genotypes to underlying genes and molecular mechanisms influencing economically important traits.

## Conclusions

The dense linkage map presented was used to outline the distribution of homeologous regions within the Atlantic salmon genome. This linkage map will be a valuable resource for addressing important aquaculture, ecological and evolutionary questions and to assist in the assembly of the forthcoming reference genome sequence for Atlantic salmon.

## Methods

### SNP-chip

A custom design Illumina iSelect SNP-array, containing approximately 6 K working SNP-assays was developed in-house at CIGENE. Approximately half of markers were identified from EST alignments [18,19], with the remainder detected following 454 sequencing of reduced representation libraries from eight individual salmon belonging to a commercial breeding population in Norway.

Markers integrated in the linkage map were generated from the following sources:

i) 2929 from sequencing of genome complexity reduction (GCR) libraries using 454 technology,

ii) 2824 from EST alignments [18,19],

iii) 124 from low-scale targeted re-sequencing of BAC-end sequences [22] and

iv) 42 from other gene related sequences.

### Classification of markers, genotyping and SNP filtering

Samples were genotyped following standard protocols for iSelect SNP-array. Bead-arrays were scanned on an iScan reader using a modified Infinium II scan settings protocol which records bead-level intensity data in .txt format. A large fraction of markers in the salmon genome showed polyploidy caused by the WGD in the early evolution of the Salmonidae family. Such markers may be classified as multisite variants or MSVs as suggested by Fredman et al. [23]. In instances where markers segregate in one of the paralogs with the other being fixed, the samples cluster into three groups depending on the (mixed) allelic ratio of the marker in the sample. These are denoted MSV-3 and can be distinguished from regular SNPs in that the clusters are much more tightly positioned. Illumina's GenomeStudio Genotyping Analysis Module (v.1.6.3) may be tuned to cluster some of the MSV-3s; however they will be regarded as regular SNPs. MSVs polymorphic in both paralogs were revealed by five separate clusters and therefore were classified as MSV-5s. Unfortunately the current version of GenomeStudio is not designed to call polyploid genotypes and consistently failed to call or mis-called MSV-5s. In order to improve genotyping efficiency of the data we developed a pipeline for validation, quality filtering and allele scoring in Atlantic salmon. The pipeline efficiently differentiates between reliable and unreliable SNP assays and improves data confidence. A key element in the pipeline is our development of the R-package *beadarrayMSV *[17] made freely available on the web at http://cran.r-project.org/.

### Family material

Atlantic salmon genomic DNA was extracted from fin-clips provided by the Norwegian breeding company Aqua Gen. Close to 3500 fish (offspring and parents) distributed among a mixture of half-sib and full-sib families were genotyped using iSelect SNP-array following standard protocols (Illumina, San Diego, CA). After genotyping families were filtered for potential pedigree errors which resulted in a final set 3297 of salmon being used for the linkage map construction.

Modified versions of the CRIMAP 2.4 software [33], which can handle larger numbers of markers segregating in complex pedigrees, were utilized for the linkage analysis. Initially the *AUTOGROUP *option of a CRIMAP version, provided by Xuelu Liu (Monsanto, Saint Louis, MO, USA), was used to place SNPs into linkage groups based on twopoint LOD scores. The SNPs were included stepwise starting with the most informative SNPs together with SNPs with a known chromosomal position from the previous studies of Moen et al. [21], Danzmann et al. [6] and Lorenz et al. [22]. After the initial grouping of SNPs to 29 linkage groups, markers were ordered within group using the *BUILD *and *FLIPSN *options of CRIMAP. Due to the extremely low recombination rate in males across the vast majority of the genome the initial order of markers were determined based on female meioses only. An in-house modified version of CRIMAP was developed to deal with the larger numbers of markers and pedigree in the analyses. Subsequently marker orders were determined in both sexes using a combination of *FLIPSN, CHROMPIC *and *FIXED *of the same program. After determining the most likely SNP-order within linkage groups double recombinants were identified by *CHROMPIC *option of CRIMAP and a script were written to correct erroneous genotypes. MSV-5 markers could not be directly mapped as they display the mean signal from two separate loci. As a consequence MSV-5 marker alleles were determined within families by *beadarrayMSV *as described by Gidskehaug et al. [17] and pooled with other SNP genotypes in a combined analysis. Sex-specific multipoint linkage maps were constructed using the *FIXED *option of CRIMAP. An R-script was written for graphical visualization of linkage groups representing 29 chromosomes in Atlantic salmon following the nomenclature of Phillips et al., [20].

### BLAST searches

SNP sequences were compared with each other using megablast in order to identify paralogous genes and identify homeologous chromosomal regions within the Atlantic salmon genome. Megablast was also used when searching for homeologous regions in Atlantic salmon that match up with conserved syntenic groups in the sequenced stickleback genome. Megablast searches were performed on a local server running the Galaxy tool (http://main.g2.bx.psu.edu/.

## Competing interests

None declared. TM is employee of AquaGen.

## Authors' contributions

SL: conceived of the study, constructed genetic maps, performed comparative analyses and drafted the manuscript. LG: MSV-5 mapping, graphical visualization of linkage maps and assisted in finalizing the manuscript. TM: Provided family material and assisted in finalizing the manuscript. BJH: SNP-detection from EST-data and assistance in drafting and finalizing the manuscript. PRB: SNP-validation and assisted in finalizing the manuscript. WSD: Discussion and assisted in finalizing the manuscript. SWO: Project support and assisted in finalizing the manuscript. MPK: SNP-chip development, genotyping, data cleaning and assistance in drafting and finalizing the manuscript. All authors read and approved of the final version.

## Supplementary Material

Additional file 1 **A total of 5918 marker were mapped to linkage groups by two point linkage analyses**. Among these, 5650 markers were integrated into the linkage map. The remaining 268 markers were not integrated due to insufficient statistical support or because they substantially expanded the multipoint linkage map. The columns in table are ID of the marker, classification of markers, Atlantic salmon chromosomes number, order of markers within each linkage group, female linkage map in cM, male linkage map in cM, number of meioses, sequence flanking the marker, length of the flanking sequence and dbSNP accession number. Click here for file

Additional file 2 **Graphical visualization of linkage maps**. The sections of the large acrocentric chromosomes proximal and distal to the central block of repetitive DNA are labeled qa and qb, respectively. The largest acrocentric chromosome pair has two blocks of repetitive DNA dividing the arm into three parts: 9qa, 9qb and 9qc. Click here for file
