# Supplementary figures and images for: A dense SNP-based linkage map for Atlantic salmon (Salmo salar) reveals extended chromosome homeologies and striking differences in sex-specific recombination patterns
Source: BMC Genomics. 2011 Dec 19;12:615. doi: 10.1186/1471-2164-12-615 (PMC3261913; doi:10.1186/1471-2164-12-615)

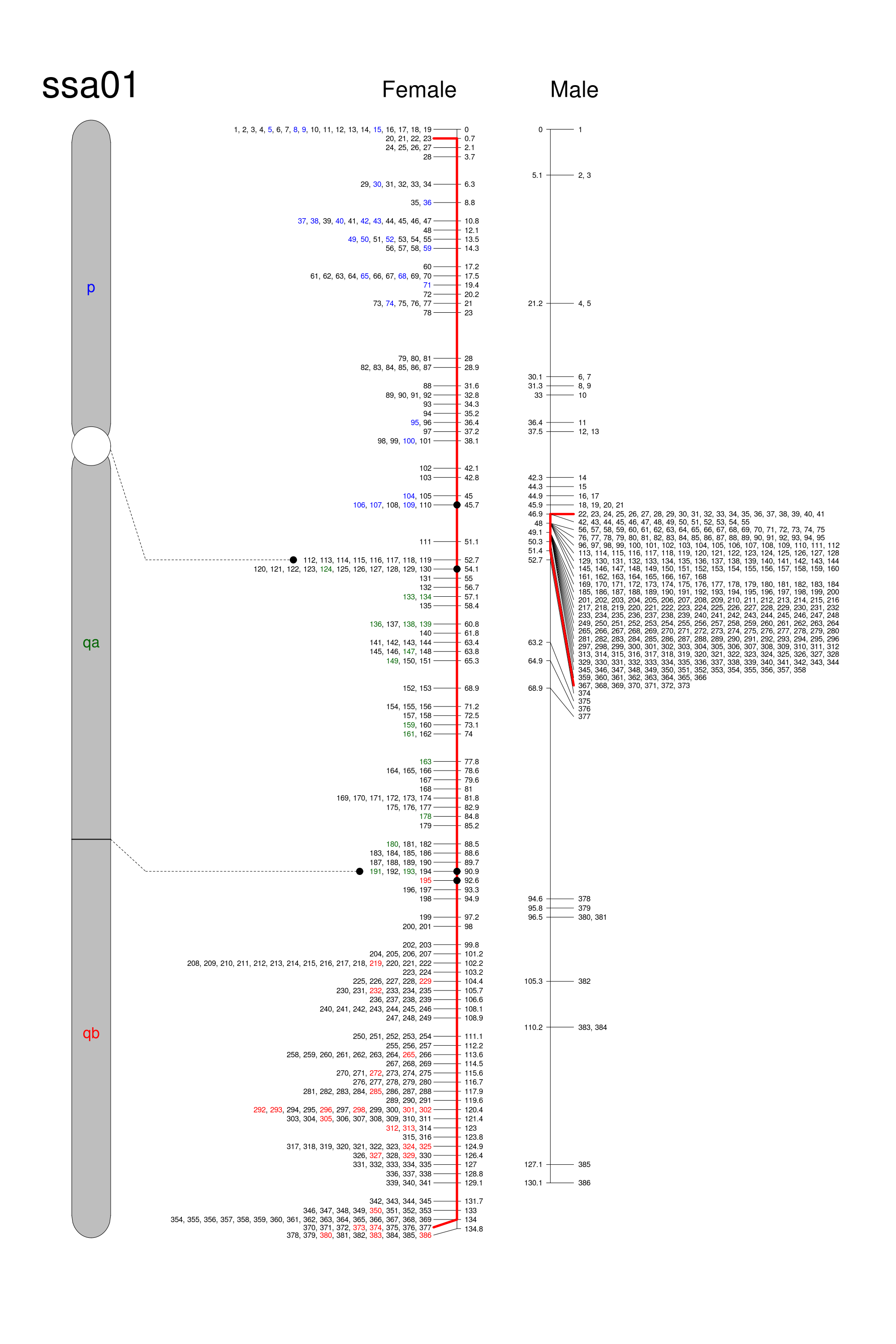

Supplement: Additional file 2 — Graphical visualization of linkage maps. The sections of the large acrocentric chromosomes proximal and distal to the central block of repetitive DNA are labeled qa and qb, respectively. The largest acrocentric chromosome pair has two blocks of repetitive DNA dividing the arm into three parts: 9qa, 9qb and 9qc. [file 1471-2164-12-615-S2.ZIP › ssa01.png]

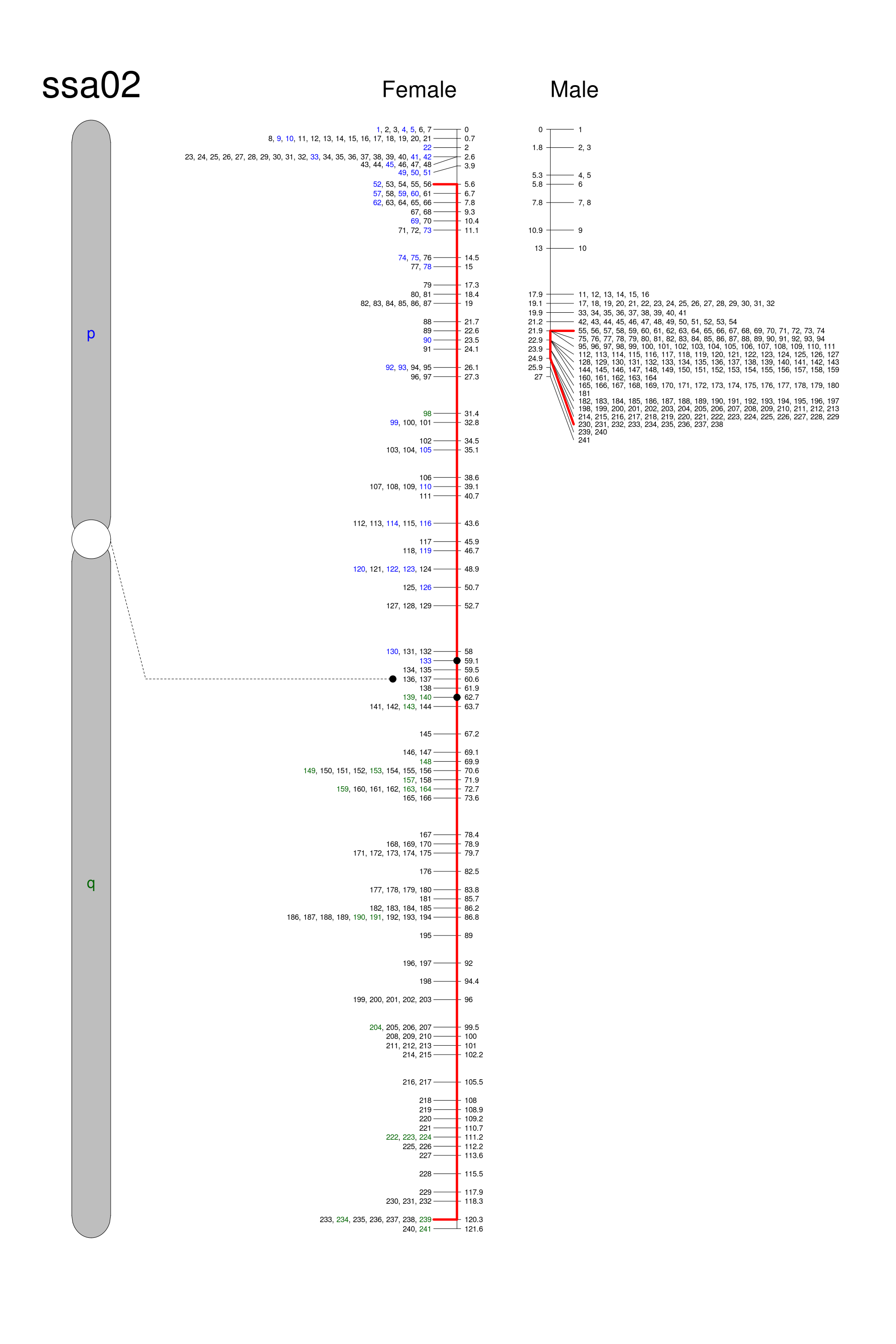

Supplement: Additional file 2 — Graphical visualization of linkage maps. The sections of the large acrocentric chromosomes proximal and distal to the central block of repetitive DNA are labeled qa and qb, respectively. The largest acrocentric chromosome pair has two blocks of repetitive DNA dividing the arm into three parts: 9qa, 9qb and 9qc. [file 1471-2164-12-615-S2.ZIP › ssa02.png]

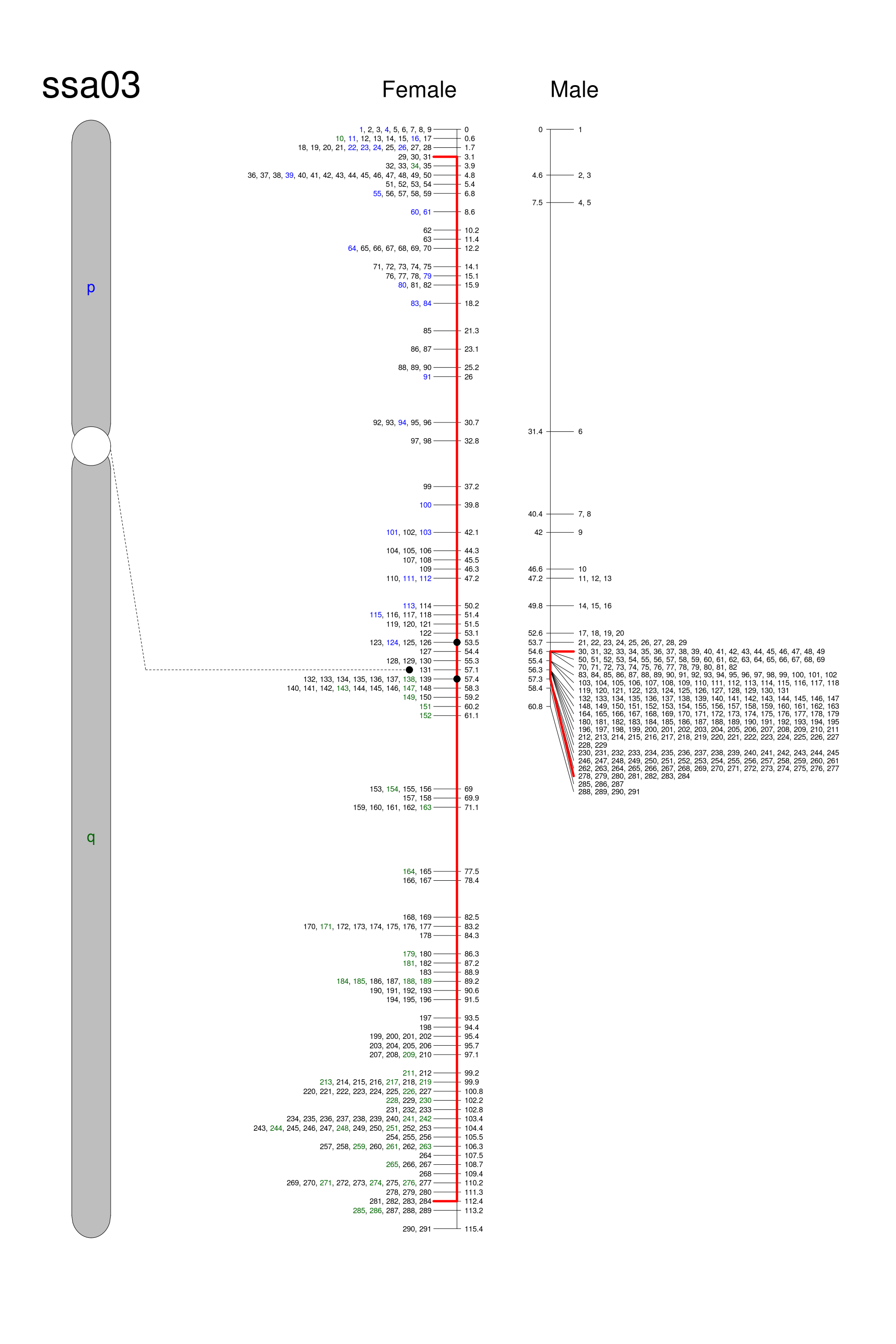

Supplement: Additional file 2 — Graphical visualization of linkage maps. The sections of the large acrocentric chromosomes proximal and distal to the central block of repetitive DNA are labeled qa and qb, respectively. The largest acrocentric chromosome pair has two blocks of repetitive DNA dividing the arm into three parts: 9qa, 9qb and 9qc. [file 1471-2164-12-615-S2.ZIP › ssa03.png]

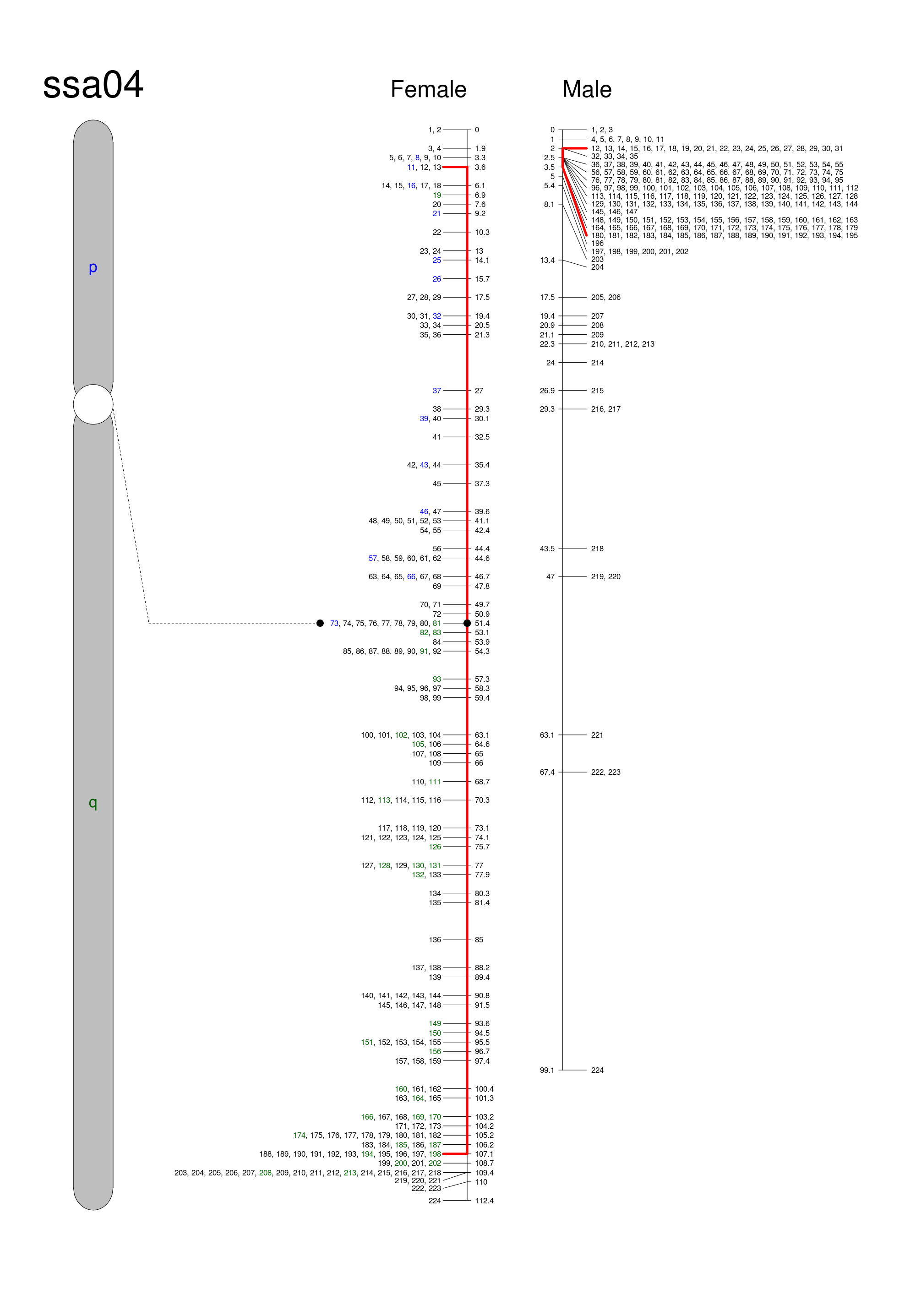

Supplement: Additional file 2 — Graphical visualization of linkage maps. The sections of the large acrocentric chromosomes proximal and distal to the central block of repetitive DNA are labeled qa and qb, respectively. The largest acrocentric chromosome pair has two blocks of repetitive DNA dividing the arm into three parts: 9qa, 9qb and 9qc. [file 1471-2164-12-615-S2.ZIP › ssa04.png]

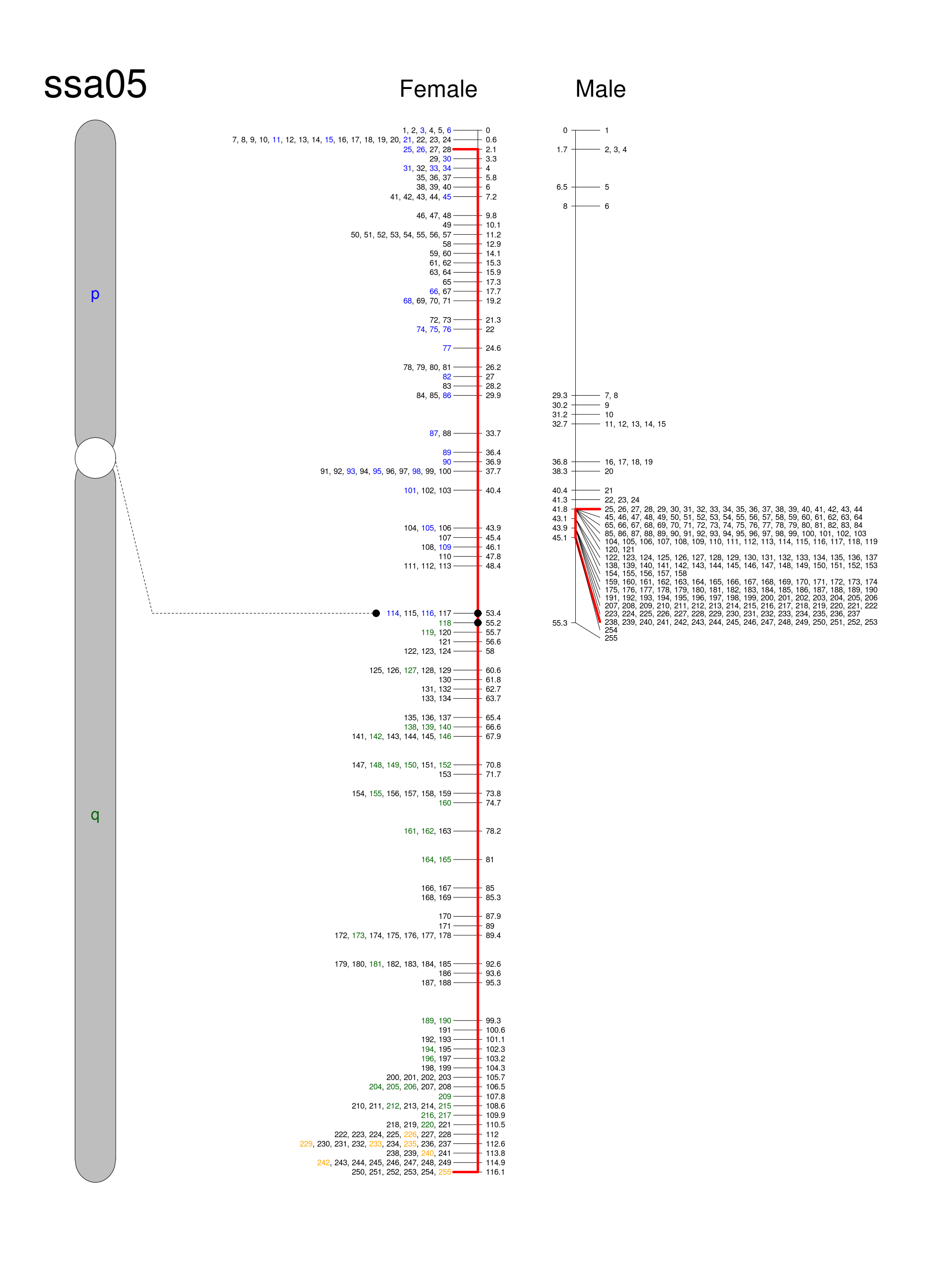

Supplement: Additional file 2 — Graphical visualization of linkage maps. The sections of the large acrocentric chromosomes proximal and distal to the central block of repetitive DNA are labeled qa and qb, respectively. The largest acrocentric chromosome pair has two blocks of repetitive DNA dividing the arm into three parts: 9qa, 9qb and 9qc. [file 1471-2164-12-615-S2.ZIP › ssa05.png]

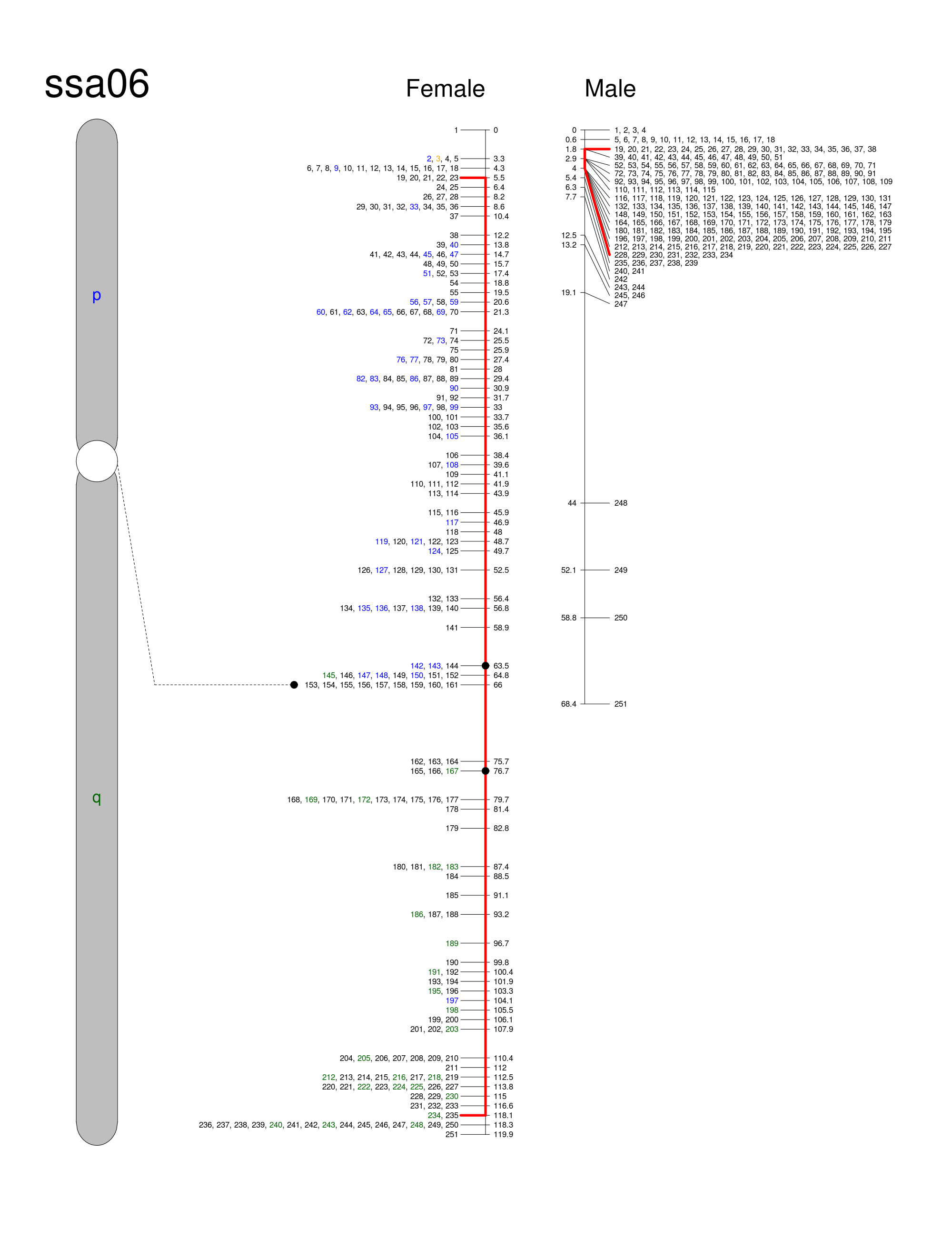

Supplement: Additional file 2 — Graphical visualization of linkage maps. The sections of the large acrocentric chromosomes proximal and distal to the central block of repetitive DNA are labeled qa and qb, respectively. The largest acrocentric chromosome pair has two blocks of repetitive DNA dividing the arm into three parts: 9qa, 9qb and 9qc. [file 1471-2164-12-615-S2.ZIP › ssa06.png]

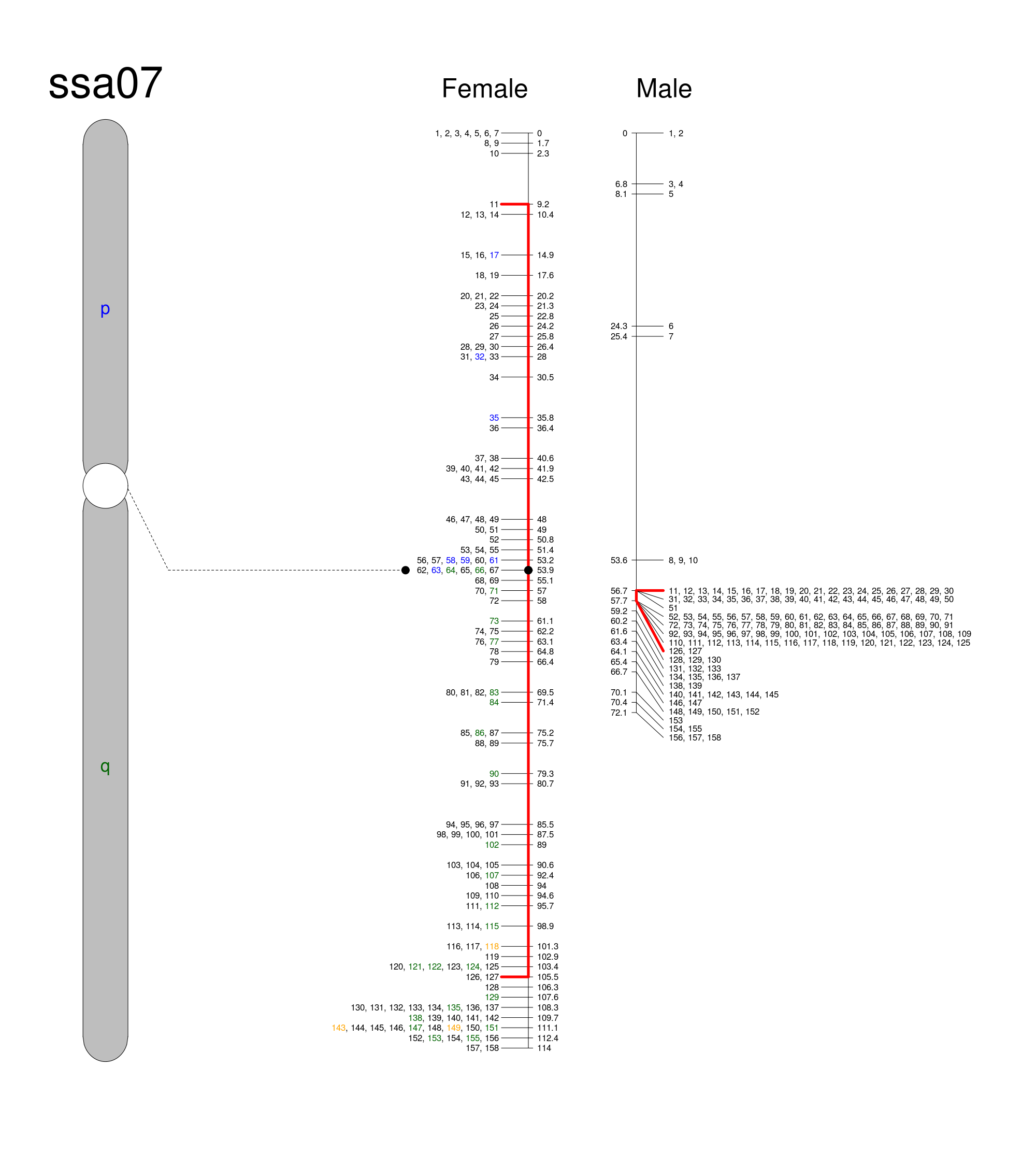

Supplement: Additional file 2 — Graphical visualization of linkage maps. The sections of the large acrocentric chromosomes proximal and distal to the central block of repetitive DNA are labeled qa and qb, respectively. The largest acrocentric chromosome pair has two blocks of repetitive DNA dividing the arm into three parts: 9qa, 9qb and 9qc. [file 1471-2164-12-615-S2.ZIP › ssa07.png]

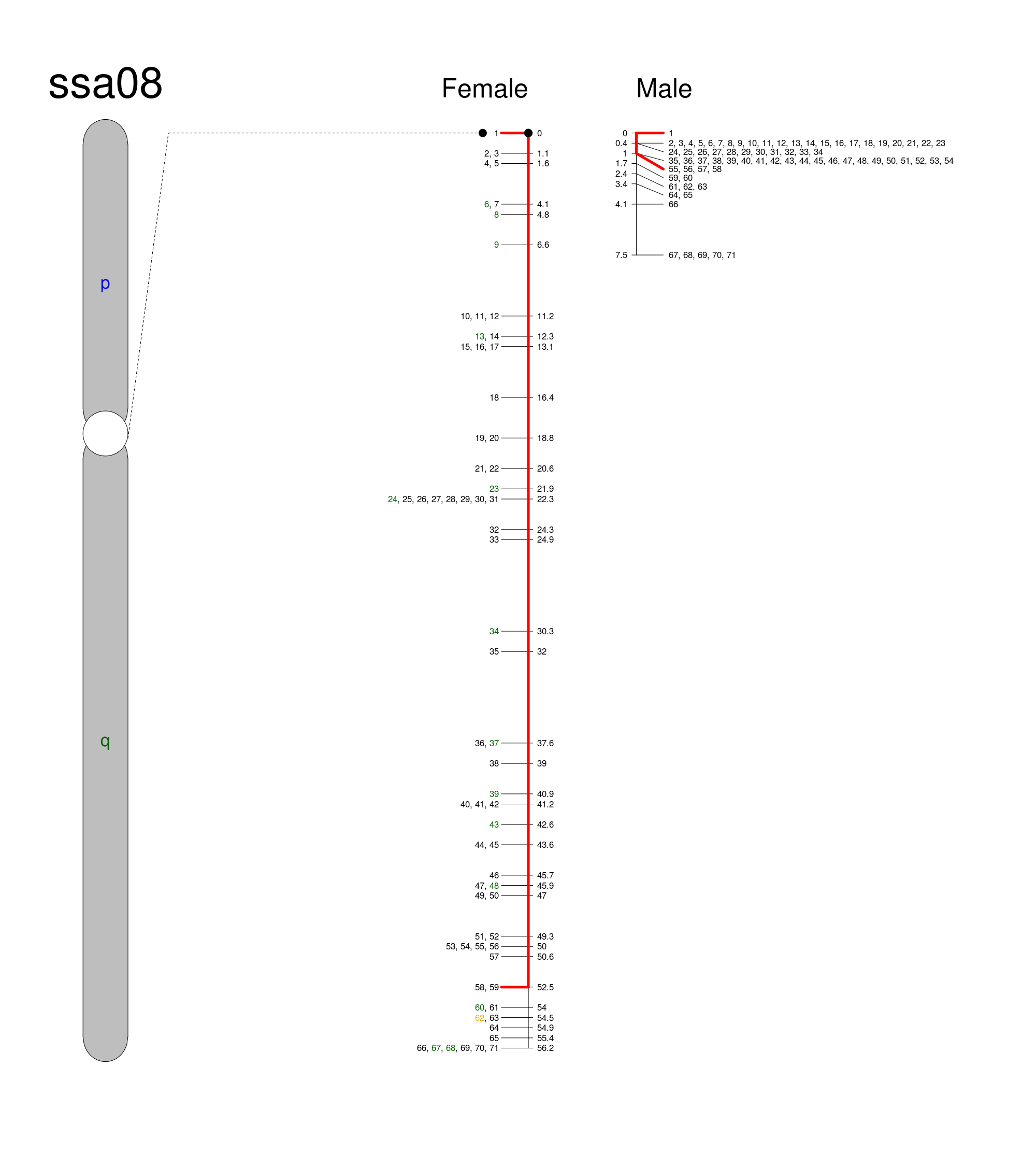

Supplement: Additional file 2 — Graphical visualization of linkage maps. The sections of the large acrocentric chromosomes proximal and distal to the central block of repetitive DNA are labeled qa and qb, respectively. The largest acrocentric chromosome pair has two blocks of repetitive DNA dividing the arm into three parts: 9qa, 9qb and 9qc. [file 1471-2164-12-615-S2.ZIP › ssa08.png]

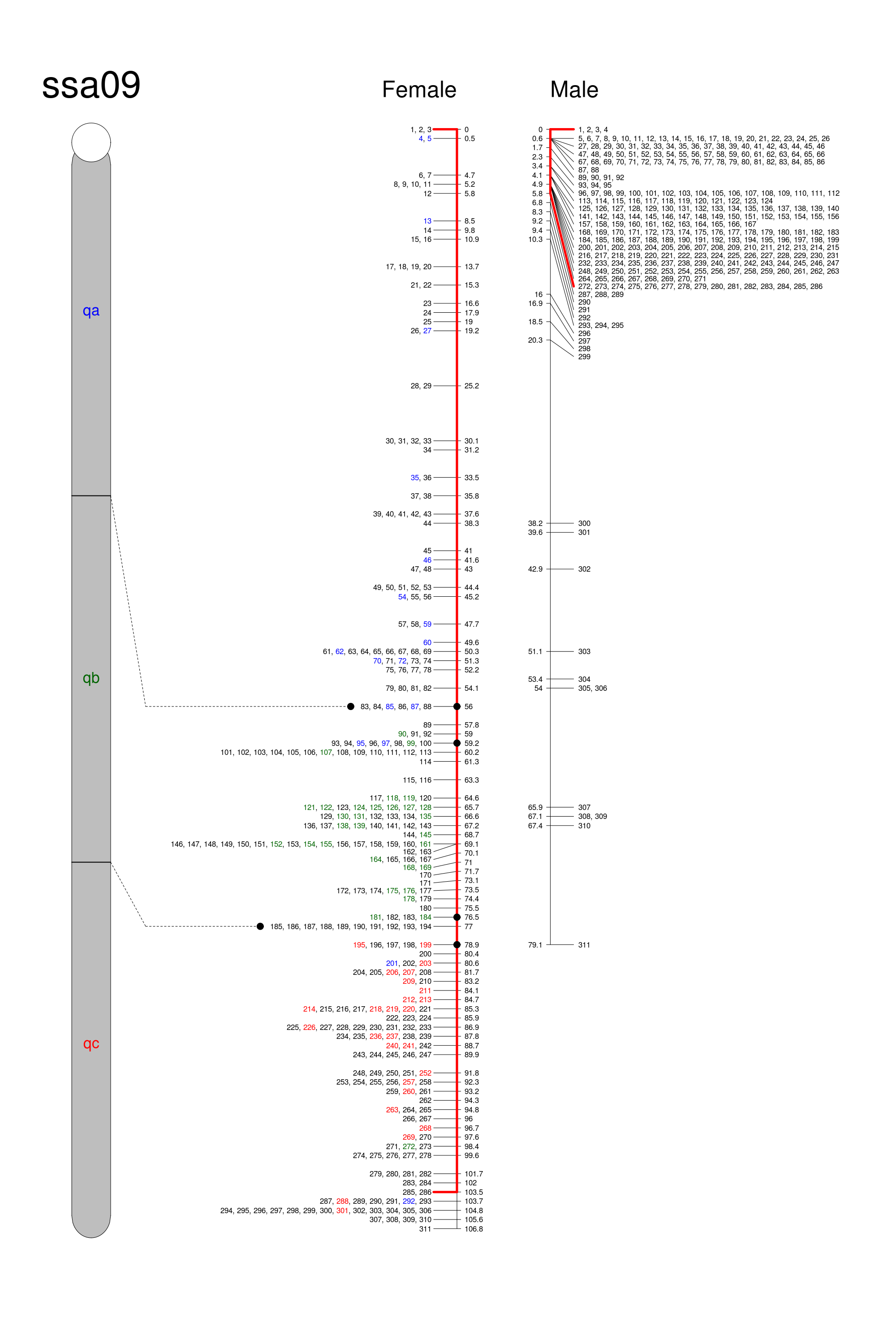

Supplement: Additional file 2 — Graphical visualization of linkage maps. The sections of the large acrocentric chromosomes proximal and distal to the central block of repetitive DNA are labeled qa and qb, respectively. The largest acrocentric chromosome pair has two blocks of repetitive DNA dividing the arm into three parts: 9qa, 9qb and 9qc. [file 1471-2164-12-615-S2.ZIP › ssa09.png]

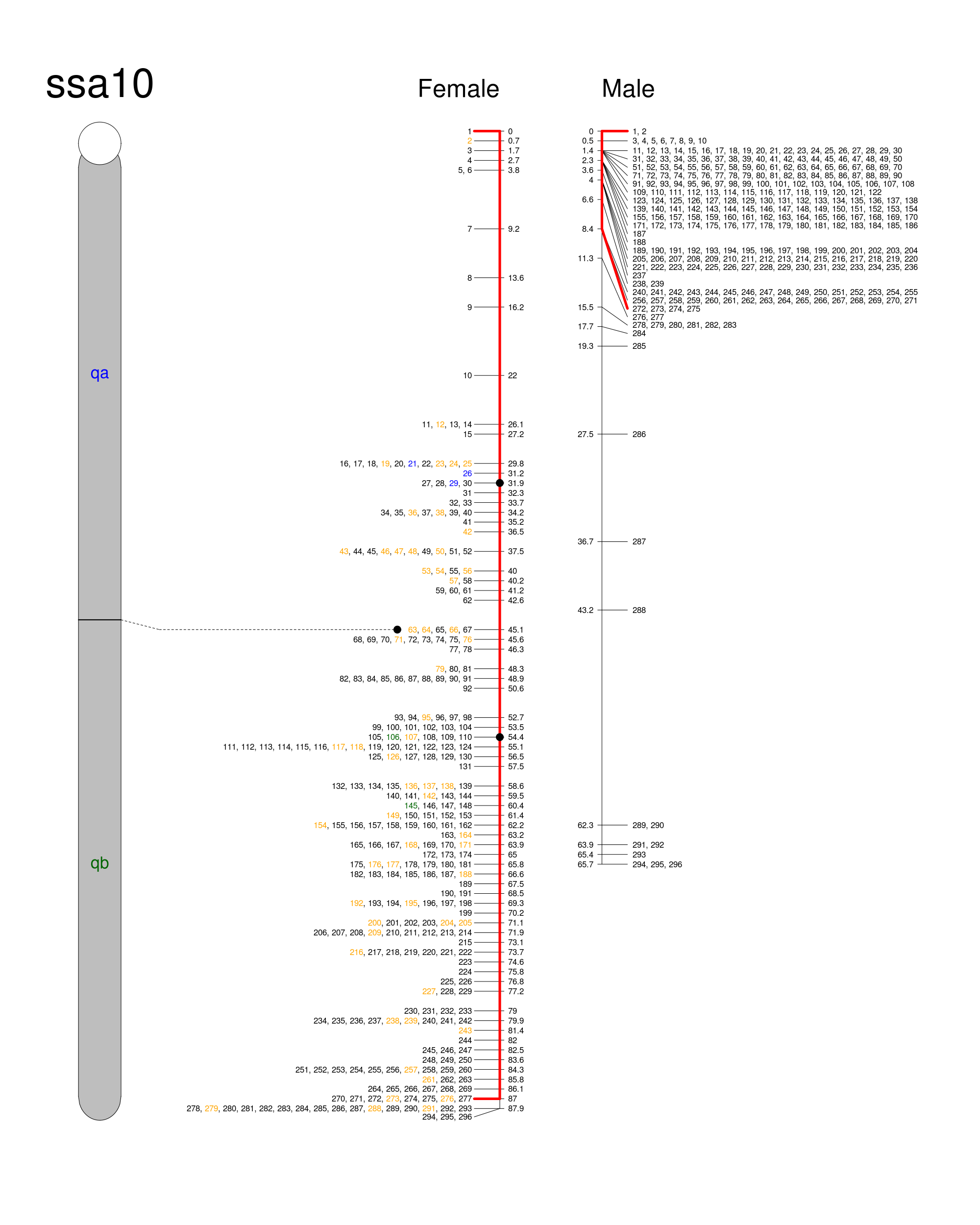

Supplement: Additional file 2 — Graphical visualization of linkage maps. The sections of the large acrocentric chromosomes proximal and distal to the central block of repetitive DNA are labeled qa and qb, respectively. The largest acrocentric chromosome pair has two blocks of repetitive DNA dividing the arm into three parts: 9qa, 9qb and 9qc. [file 1471-2164-12-615-S2.ZIP › ssa10.png]

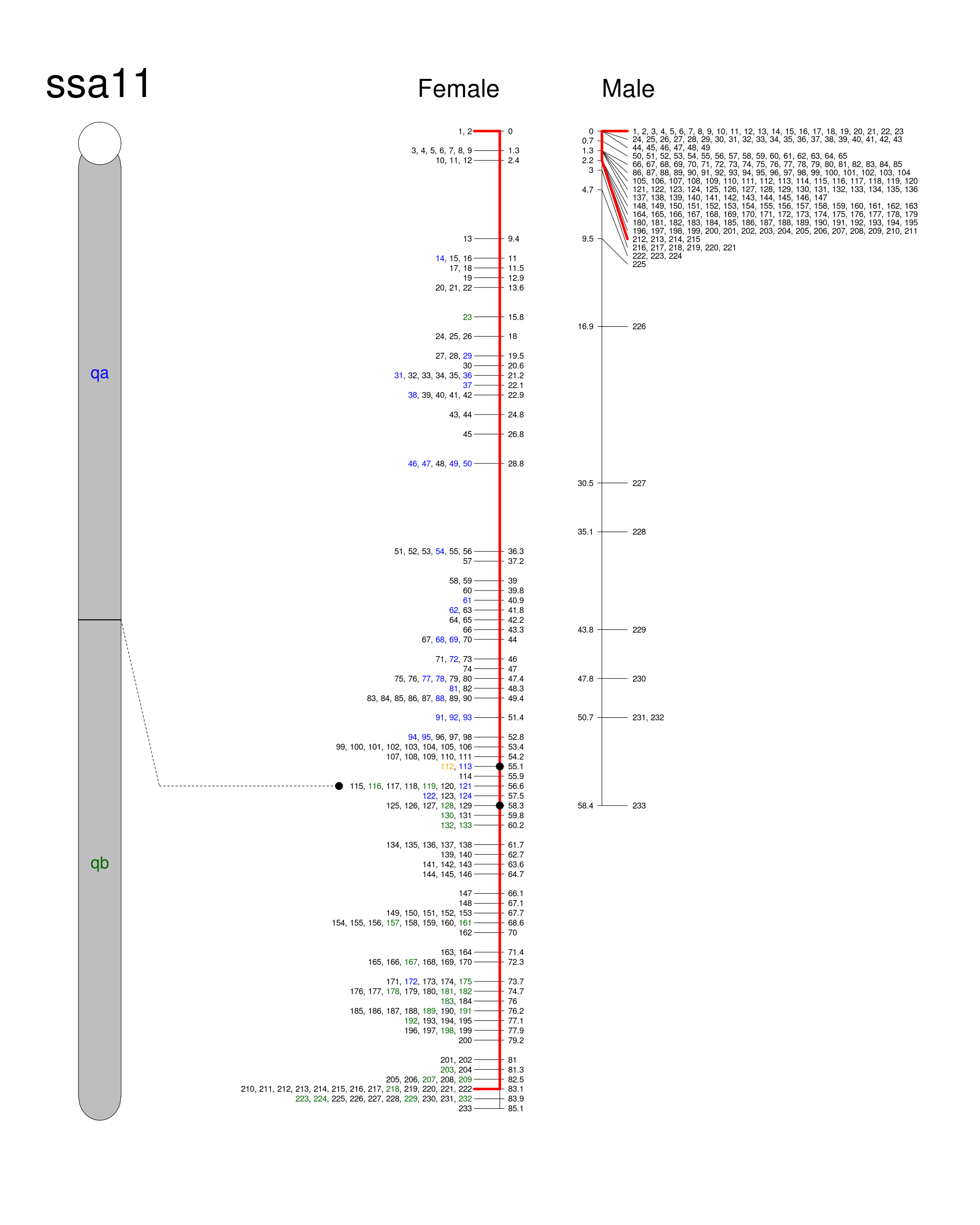

Supplement: Additional file 2 — Graphical visualization of linkage maps. The sections of the large acrocentric chromosomes proximal and distal to the central block of repetitive DNA are labeled qa and qb, respectively. The largest acrocentric chromosome pair has two blocks of repetitive DNA dividing the arm into three parts: 9qa, 9qb and 9qc. [file 1471-2164-12-615-S2.ZIP › ssa11.png]

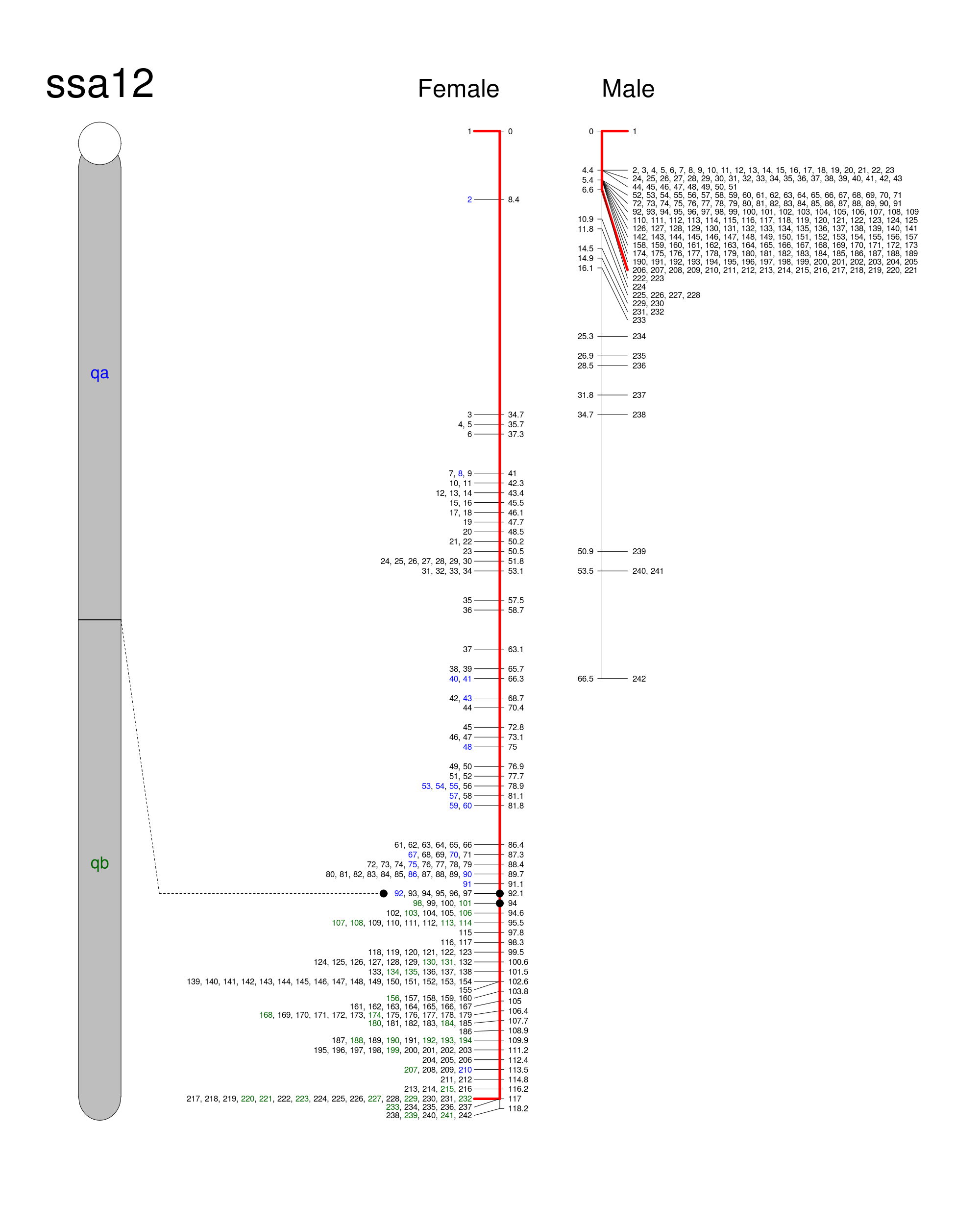

Supplement: Additional file 2 — Graphical visualization of linkage maps. The sections of the large acrocentric chromosomes proximal and distal to the central block of repetitive DNA are labeled qa and qb, respectively. The largest acrocentric chromosome pair has two blocks of repetitive DNA dividing the arm into three parts: 9qa, 9qb and 9qc. [file 1471-2164-12-615-S2.ZIP › ssa12.png]

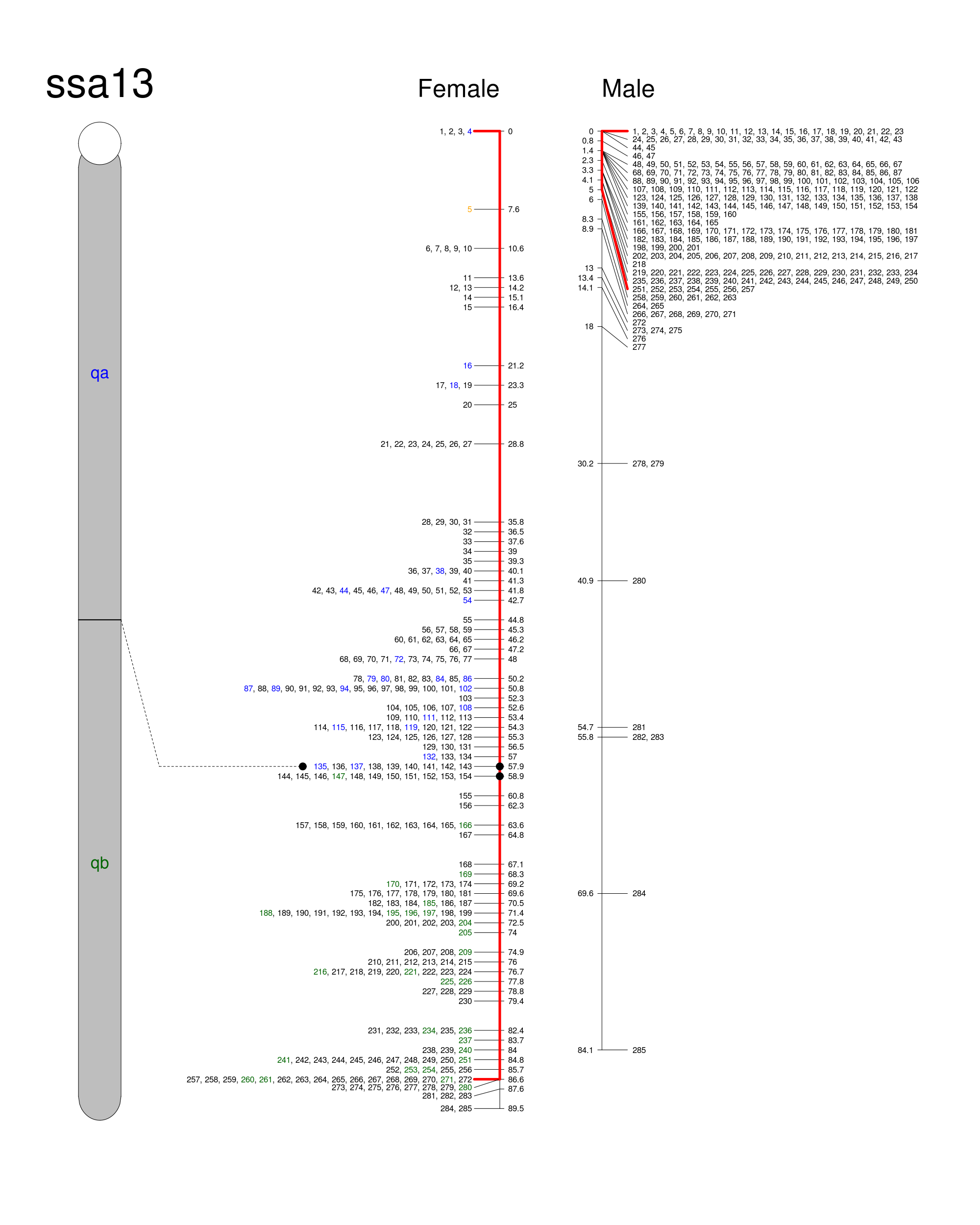

Supplement: Additional file 2 — Graphical visualization of linkage maps. The sections of the large acrocentric chromosomes proximal and distal to the central block of repetitive DNA are labeled qa and qb, respectively. The largest acrocentric chromosome pair has two blocks of repetitive DNA dividing the arm into three parts: 9qa, 9qb and 9qc. [file 1471-2164-12-615-S2.ZIP › ssa13.png]

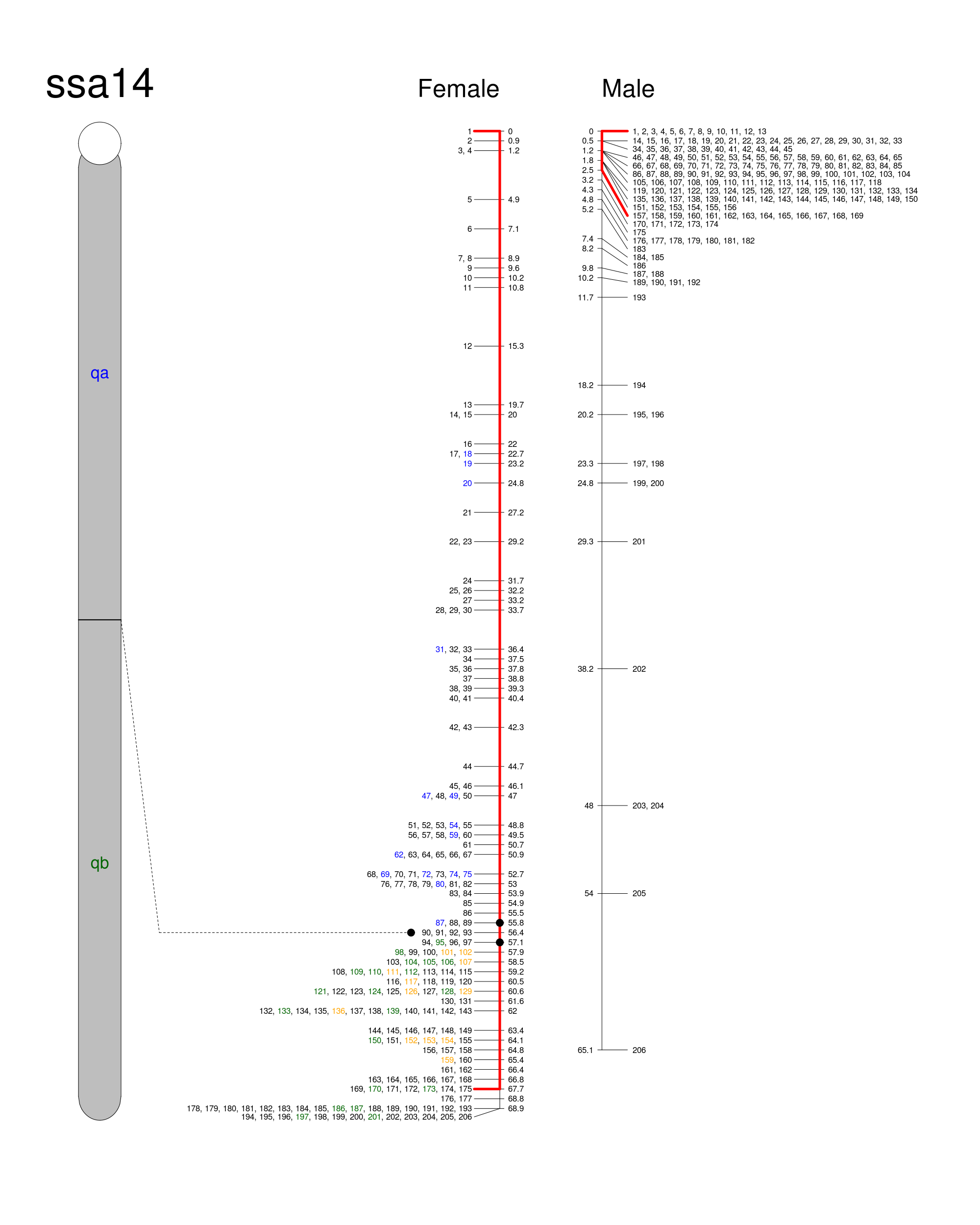

Supplement: Additional file 2 — Graphical visualization of linkage maps. The sections of the large acrocentric chromosomes proximal and distal to the central block of repetitive DNA are labeled qa and qb, respectively. The largest acrocentric chromosome pair has two blocks of repetitive DNA dividing the arm into three parts: 9qa, 9qb and 9qc. [file 1471-2164-12-615-S2.ZIP › ssa14.png]

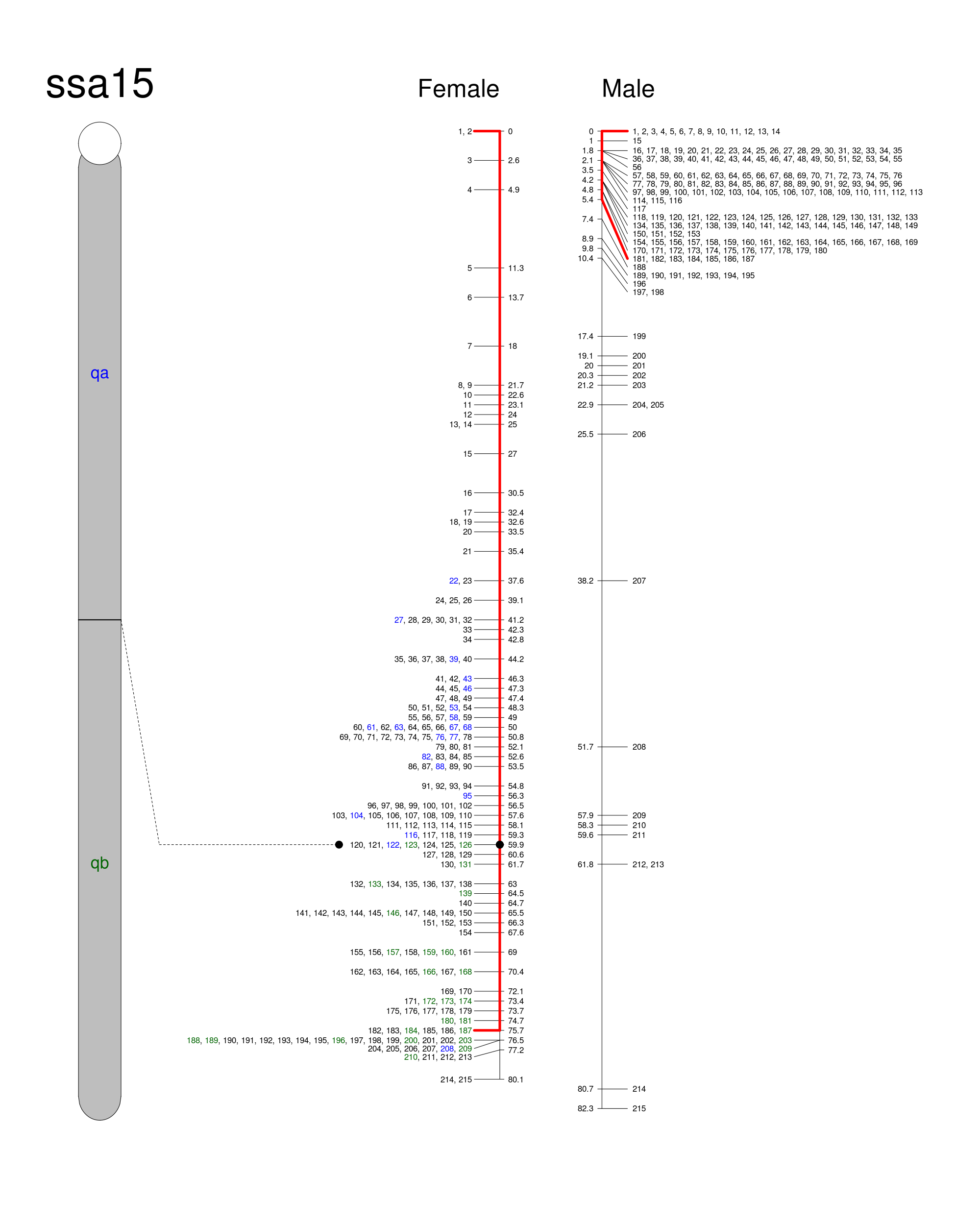

Supplement: Additional file 2 — Graphical visualization of linkage maps. The sections of the large acrocentric chromosomes proximal and distal to the central block of repetitive DNA are labeled qa and qb, respectively. The largest acrocentric chromosome pair has two blocks of repetitive DNA dividing the arm into three parts: 9qa, 9qb and 9qc. [file 1471-2164-12-615-S2.ZIP › ssa15.png]

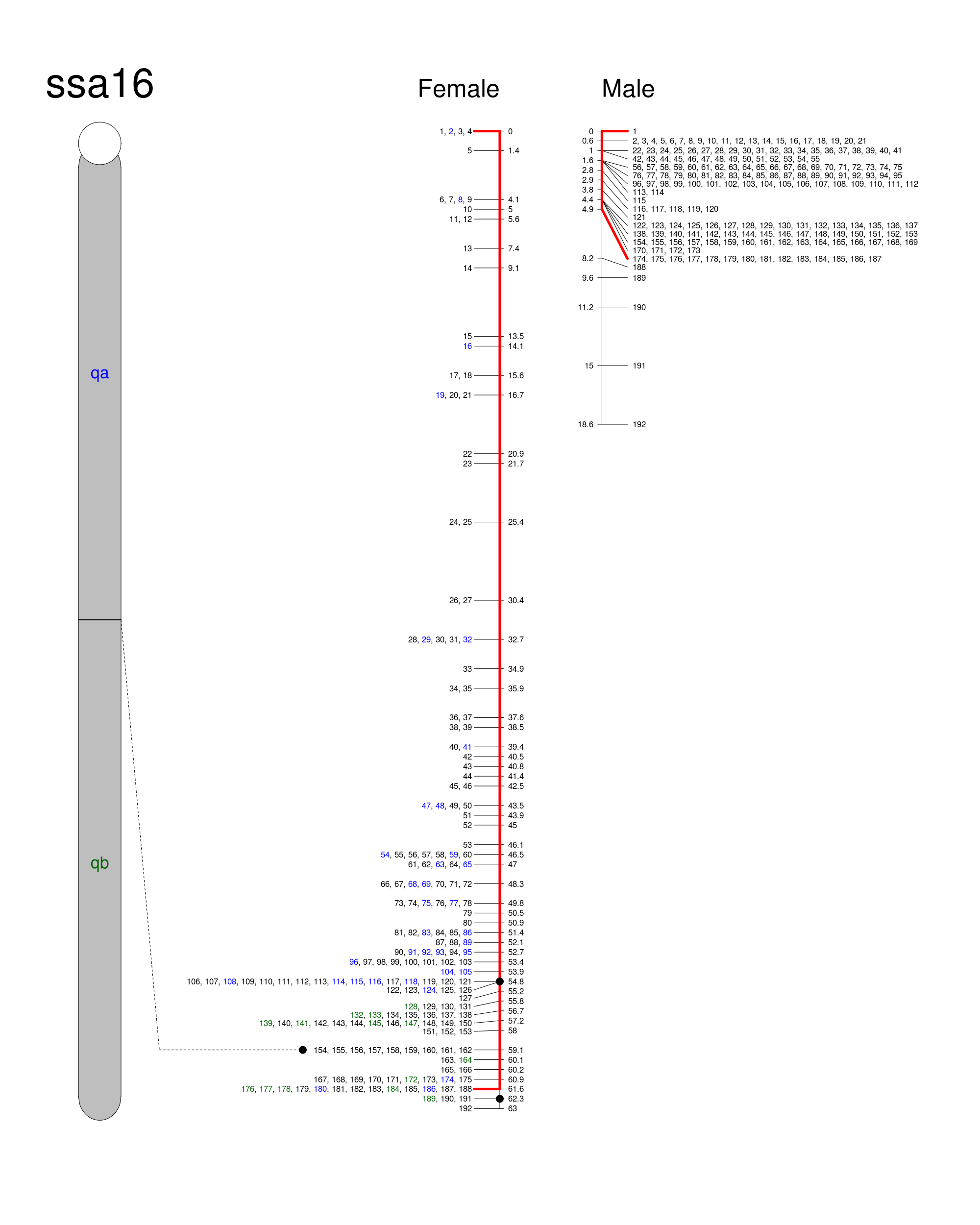

Supplement: Additional file 2 — Graphical visualization of linkage maps. The sections of the large acrocentric chromosomes proximal and distal to the central block of repetitive DNA are labeled qa and qb, respectively. The largest acrocentric chromosome pair has two blocks of repetitive DNA dividing the arm into three parts: 9qa, 9qb and 9qc. [file 1471-2164-12-615-S2.ZIP › ssa16.png]

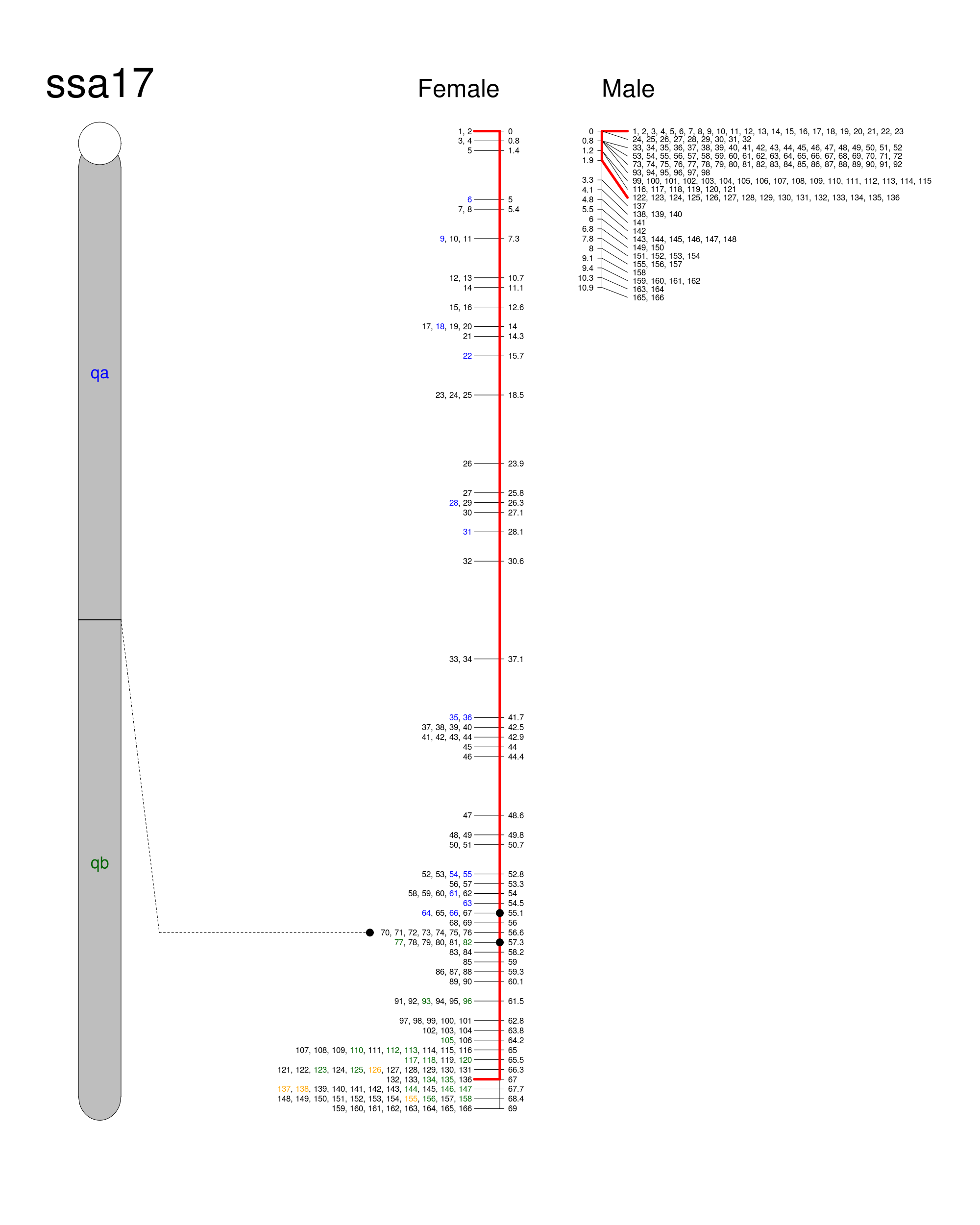

Supplement: Additional file 2 — Graphical visualization of linkage maps. The sections of the large acrocentric chromosomes proximal and distal to the central block of repetitive DNA are labeled qa and qb, respectively. The largest acrocentric chromosome pair has two blocks of repetitive DNA dividing the arm into three parts: 9qa, 9qb and 9qc. [file 1471-2164-12-615-S2.ZIP › ssa17.png]

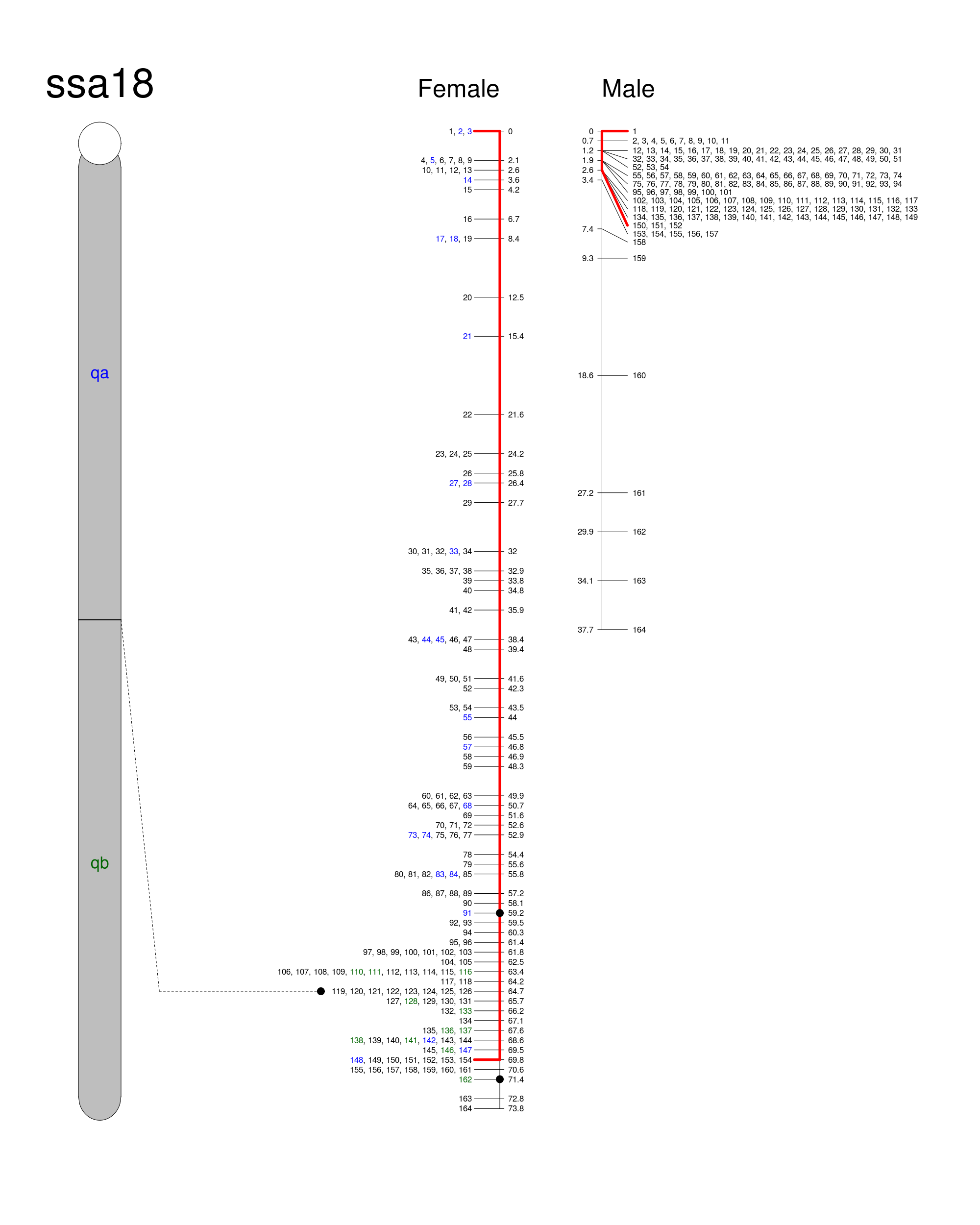

Supplement: Additional file 2 — Graphical visualization of linkage maps. The sections of the large acrocentric chromosomes proximal and distal to the central block of repetitive DNA are labeled qa and qb, respectively. The largest acrocentric chromosome pair has two blocks of repetitive DNA dividing the arm into three parts: 9qa, 9qb and 9qc. [file 1471-2164-12-615-S2.ZIP › ssa18.png]

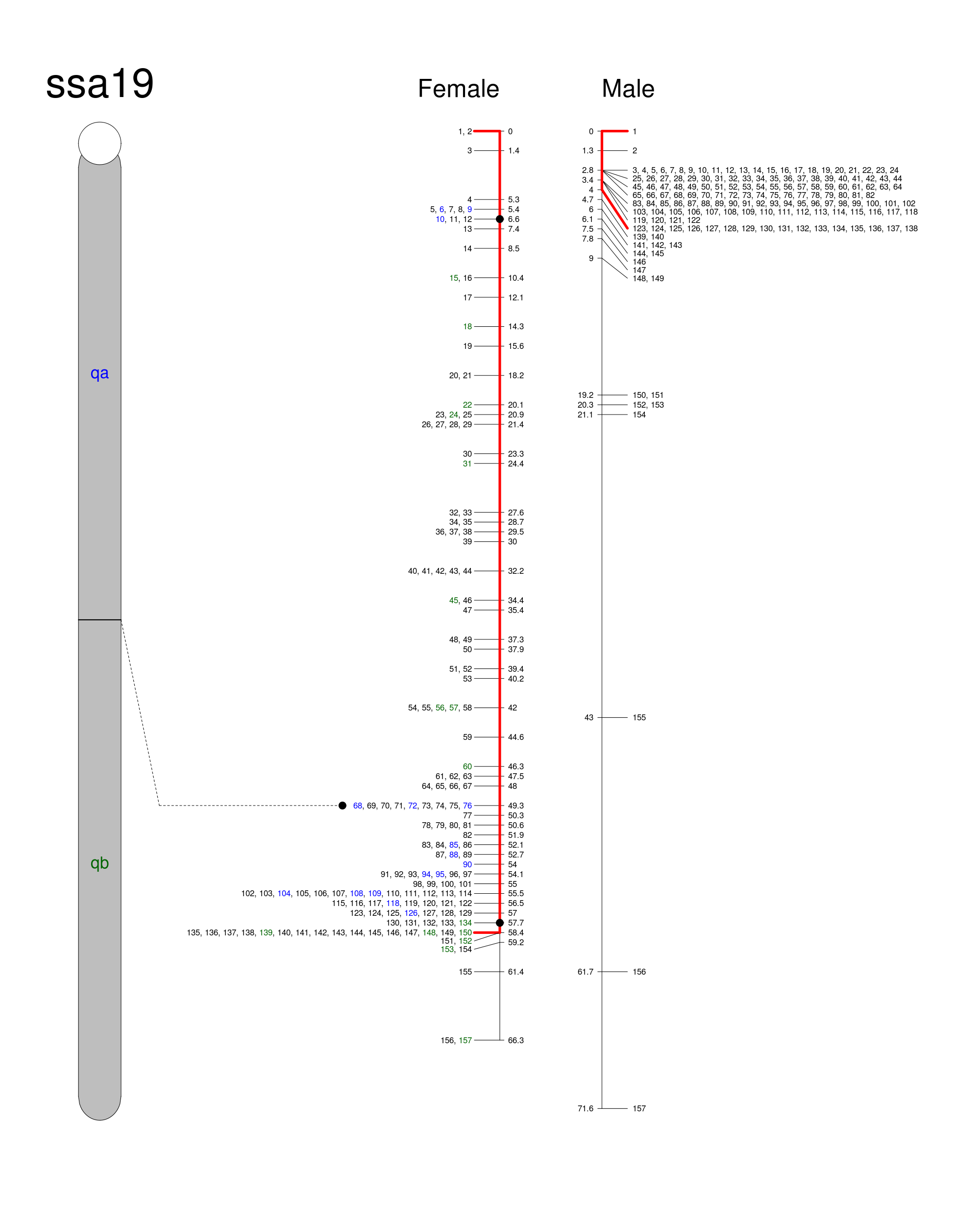

Supplement: Additional file 2 — Graphical visualization of linkage maps. The sections of the large acrocentric chromosomes proximal and distal to the central block of repetitive DNA are labeled qa and qb, respectively. The largest acrocentric chromosome pair has two blocks of repetitive DNA dividing the arm into three parts: 9qa, 9qb and 9qc. [file 1471-2164-12-615-S2.ZIP › ssa19.png]

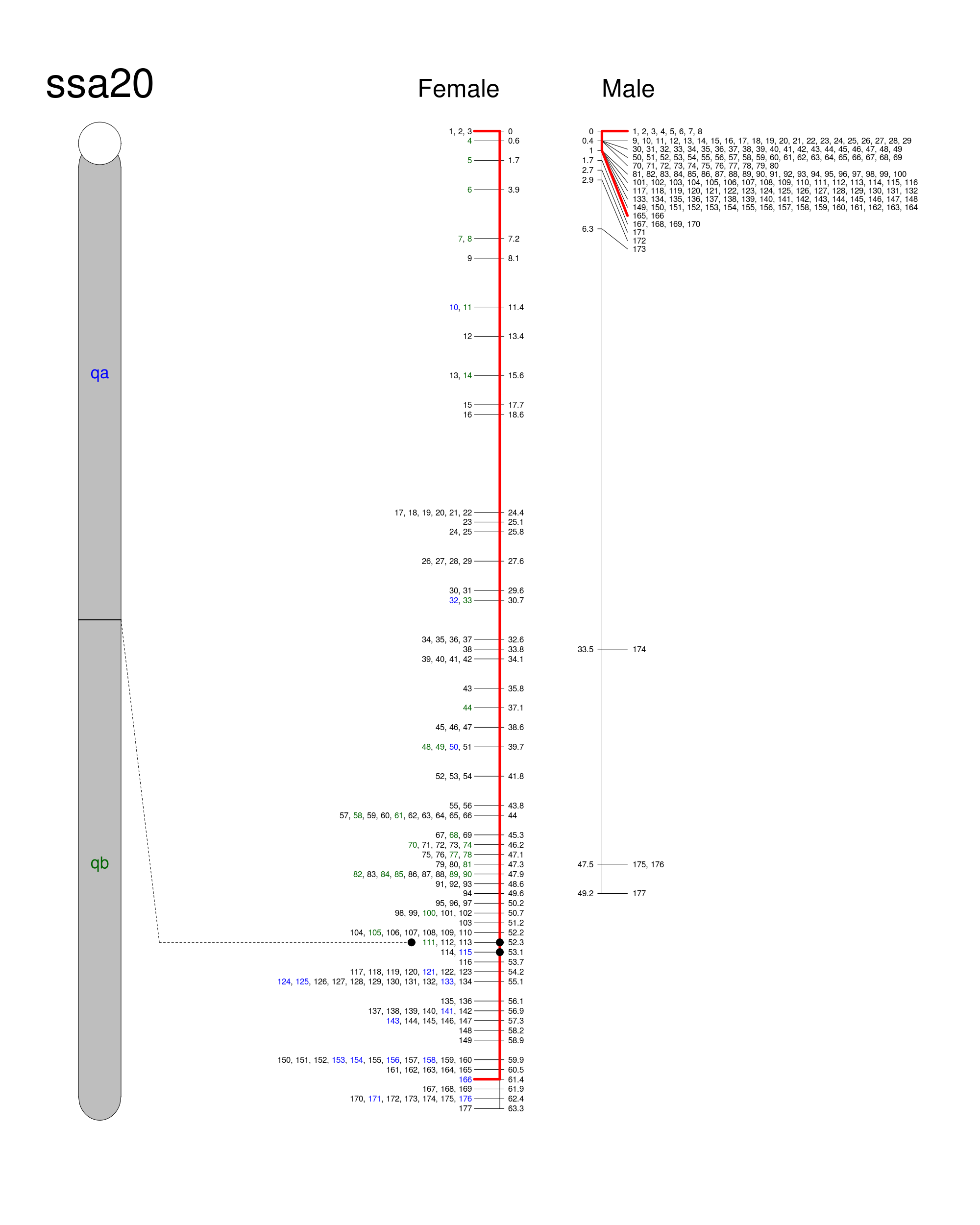

Supplement: Additional file 2 — Graphical visualization of linkage maps. The sections of the large acrocentric chromosomes proximal and distal to the central block of repetitive DNA are labeled qa and qb, respectively. The largest acrocentric chromosome pair has two blocks of repetitive DNA dividing the arm into three parts: 9qa, 9qb and 9qc. [file 1471-2164-12-615-S2.ZIP › ssa20.png]

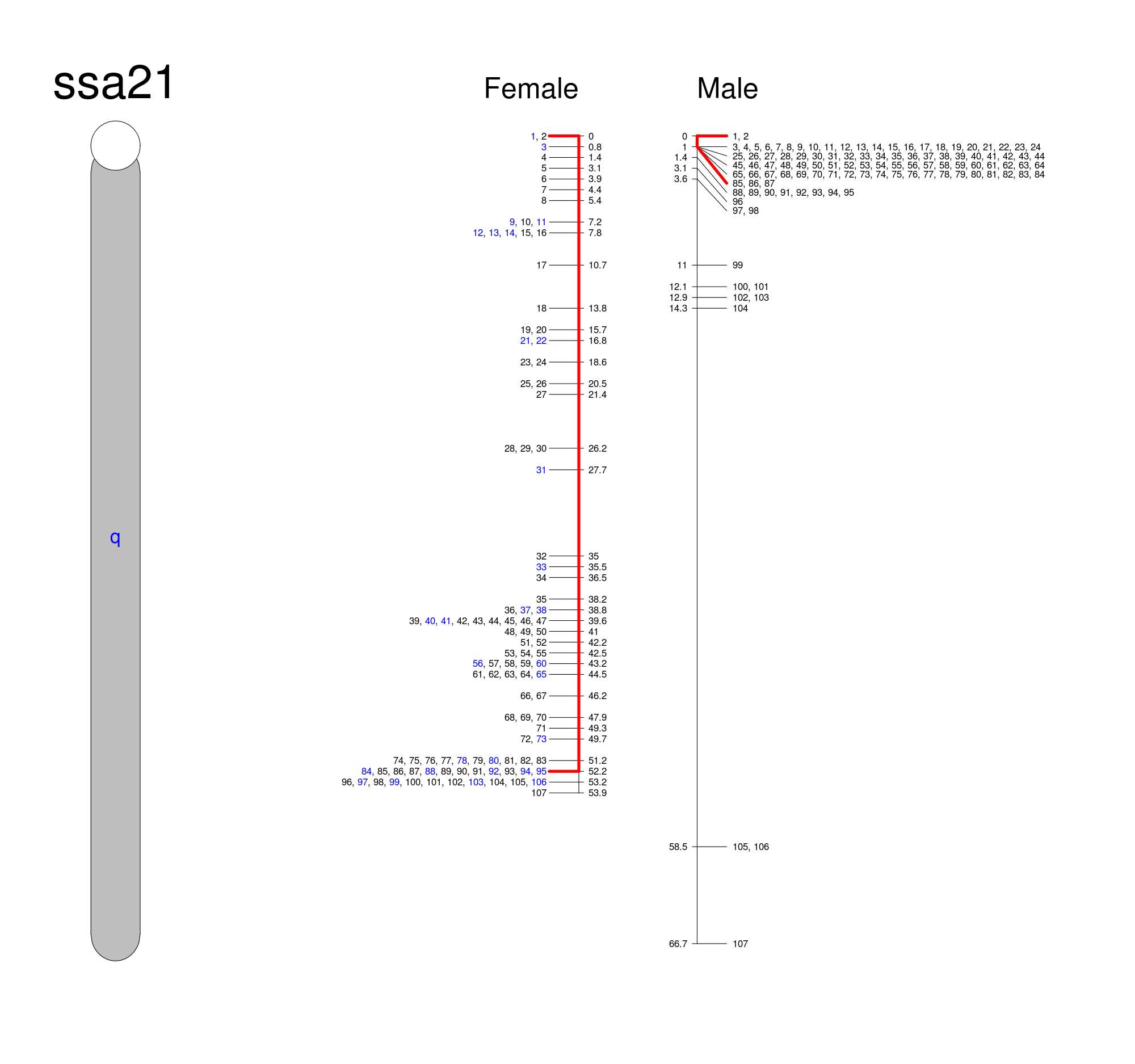

Supplement: Additional file 2 — Graphical visualization of linkage maps. The sections of the large acrocentric chromosomes proximal and distal to the central block of repetitive DNA are labeled qa and qb, respectively. The largest acrocentric chromosome pair has two blocks of repetitive DNA dividing the arm into three parts: 9qa, 9qb and 9qc. [file 1471-2164-12-615-S2.ZIP › ssa21.png]

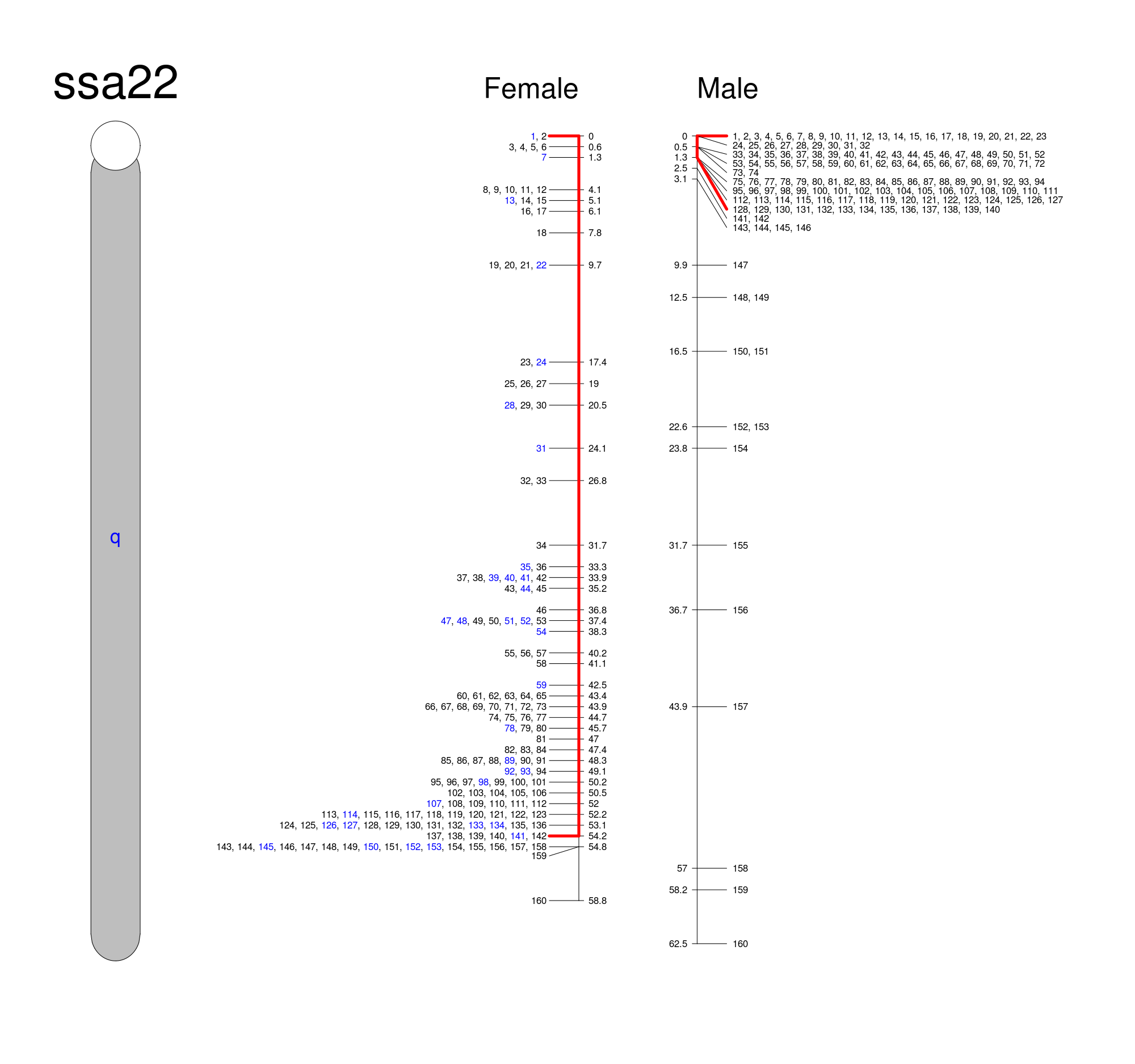

Supplement: Additional file 2 — Graphical visualization of linkage maps. The sections of the large acrocentric chromosomes proximal and distal to the central block of repetitive DNA are labeled qa and qb, respectively. The largest acrocentric chromosome pair has two blocks of repetitive DNA dividing the arm into three parts: 9qa, 9qb and 9qc. [file 1471-2164-12-615-S2.ZIP › ssa22.png]

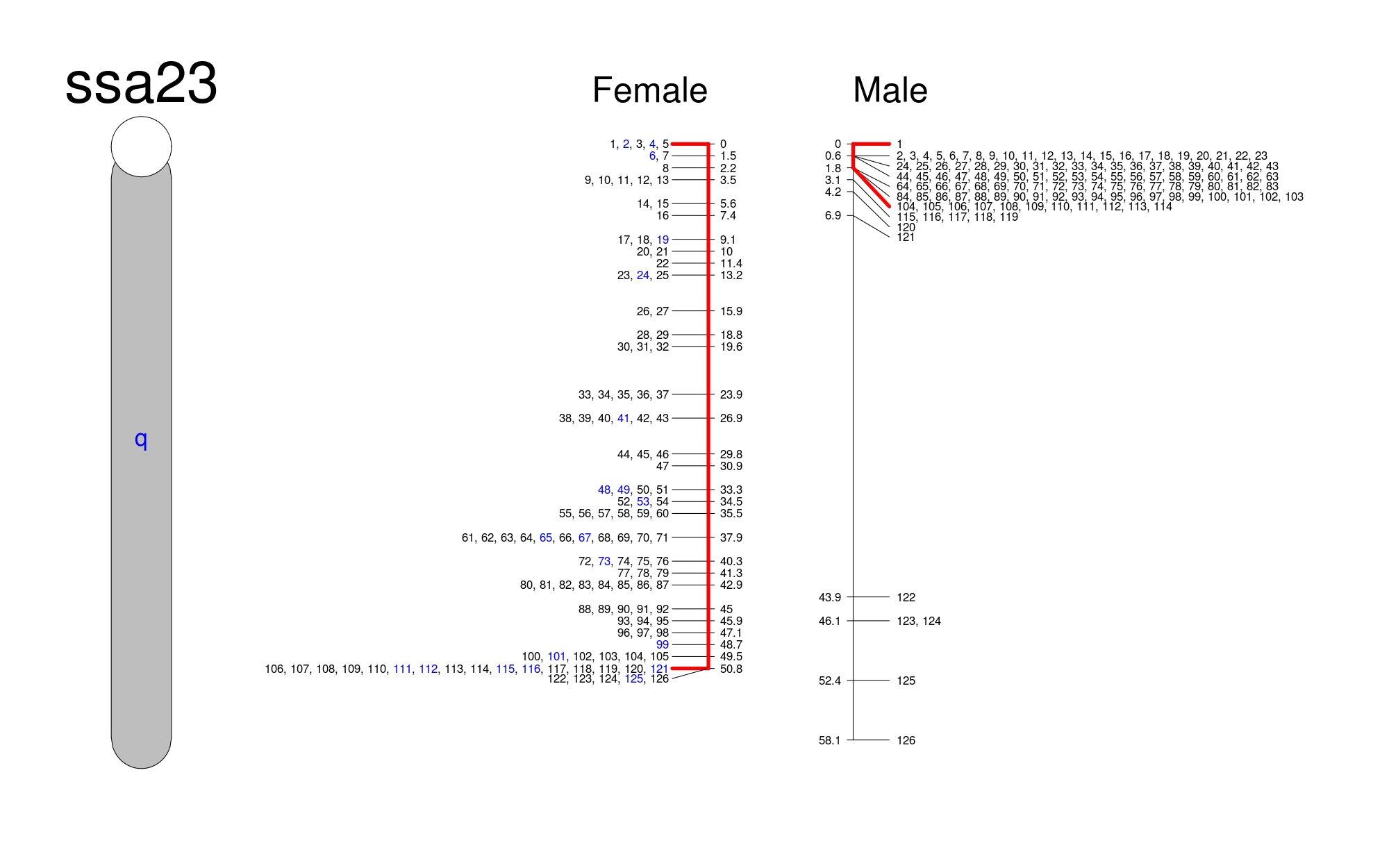

Supplement: Additional file 2 — Graphical visualization of linkage maps. The sections of the large acrocentric chromosomes proximal and distal to the central block of repetitive DNA are labeled qa and qb, respectively. The largest acrocentric chromosome pair has two blocks of repetitive DNA dividing the arm into three parts: 9qa, 9qb and 9qc. [file 1471-2164-12-615-S2.ZIP › ssa23.png]

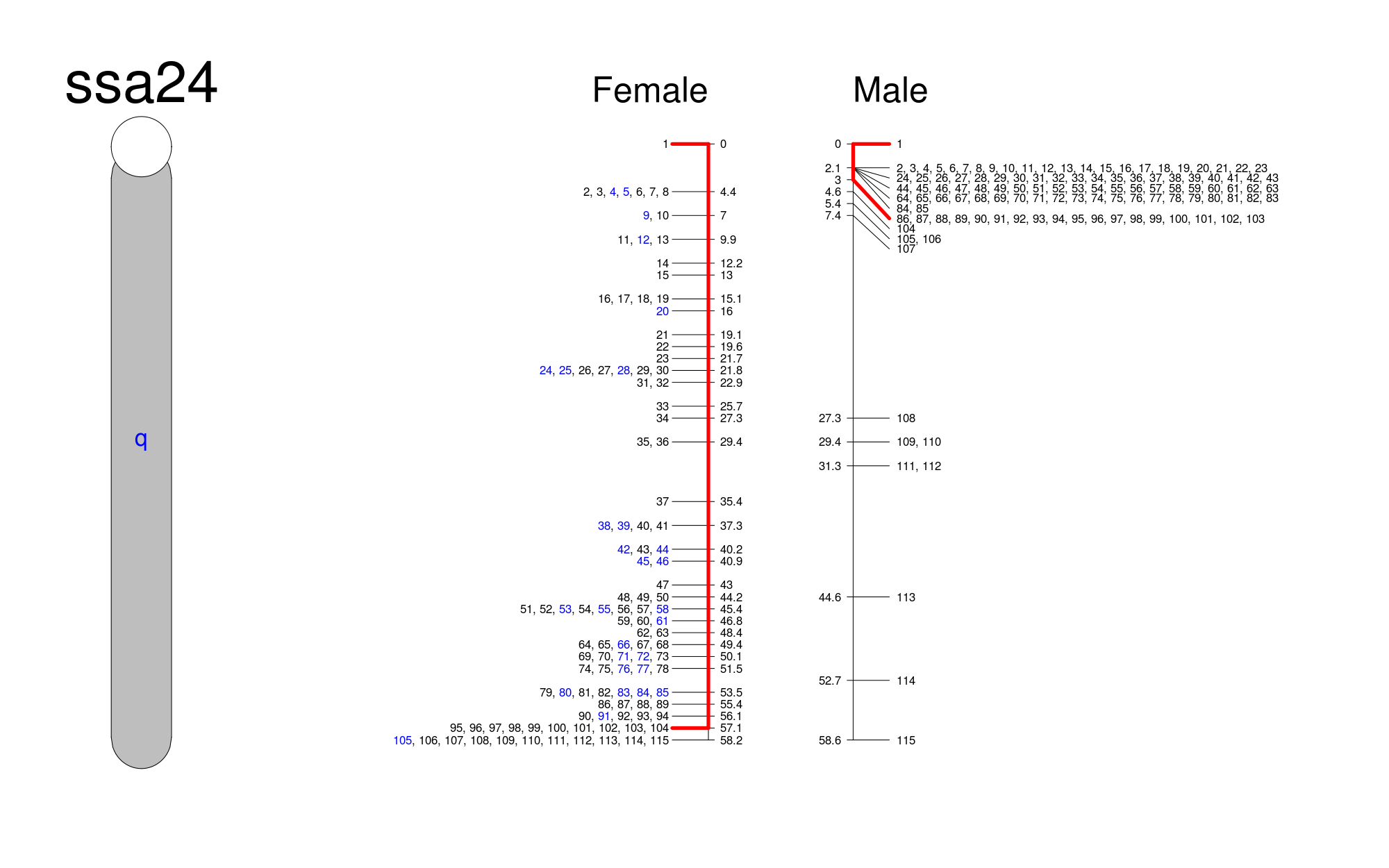

Supplement: Additional file 2 — Graphical visualization of linkage maps. The sections of the large acrocentric chromosomes proximal and distal to the central block of repetitive DNA are labeled qa and qb, respectively. The largest acrocentric chromosome pair has two blocks of repetitive DNA dividing the arm into three parts: 9qa, 9qb and 9qc. [file 1471-2164-12-615-S2.ZIP › ssa24.png]

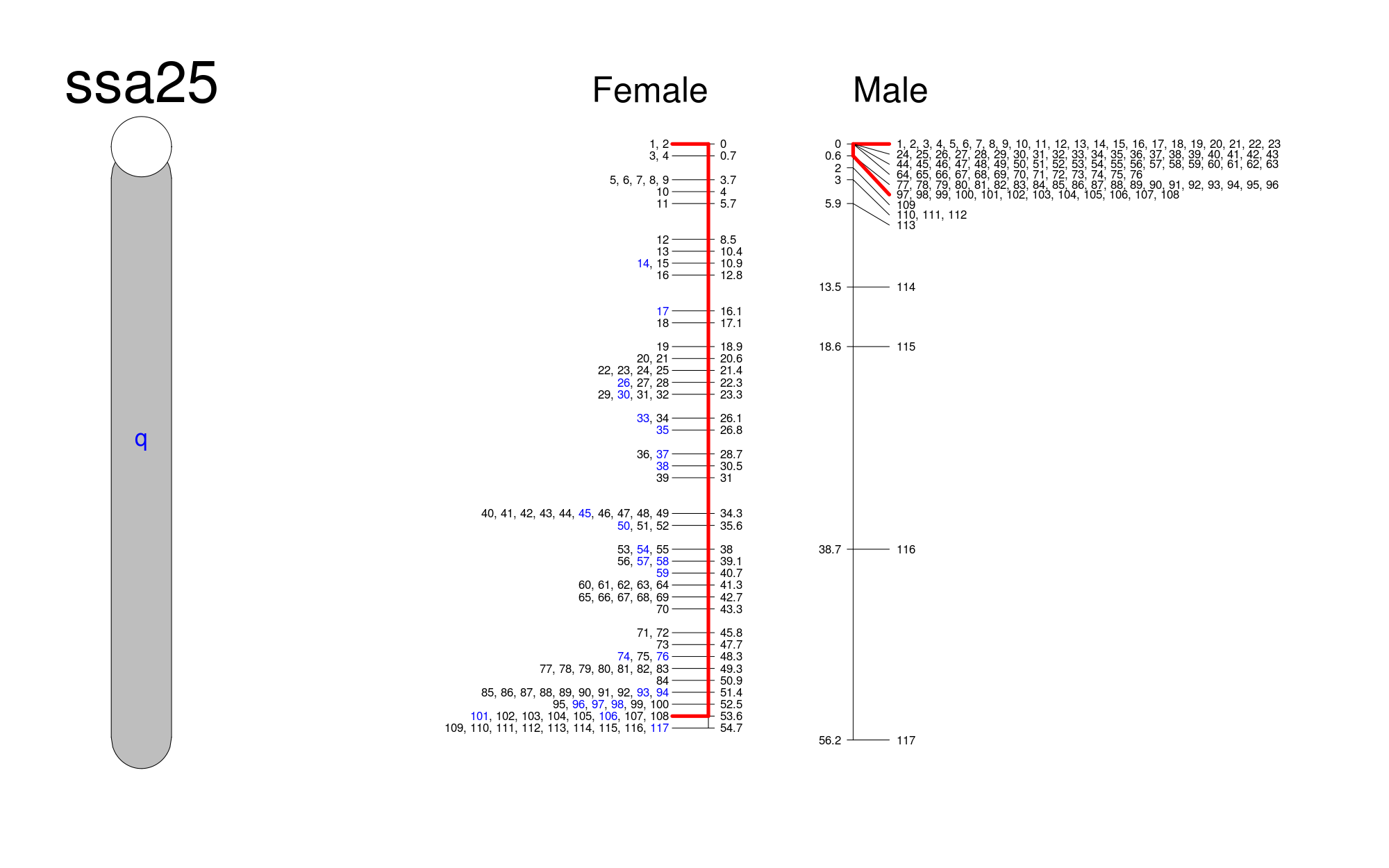

Supplement: Additional file 2 — Graphical visualization of linkage maps. The sections of the large acrocentric chromosomes proximal and distal to the central block of repetitive DNA are labeled qa and qb, respectively. The largest acrocentric chromosome pair has two blocks of repetitive DNA dividing the arm into three parts: 9qa, 9qb and 9qc. [file 1471-2164-12-615-S2.ZIP › ssa25.png]

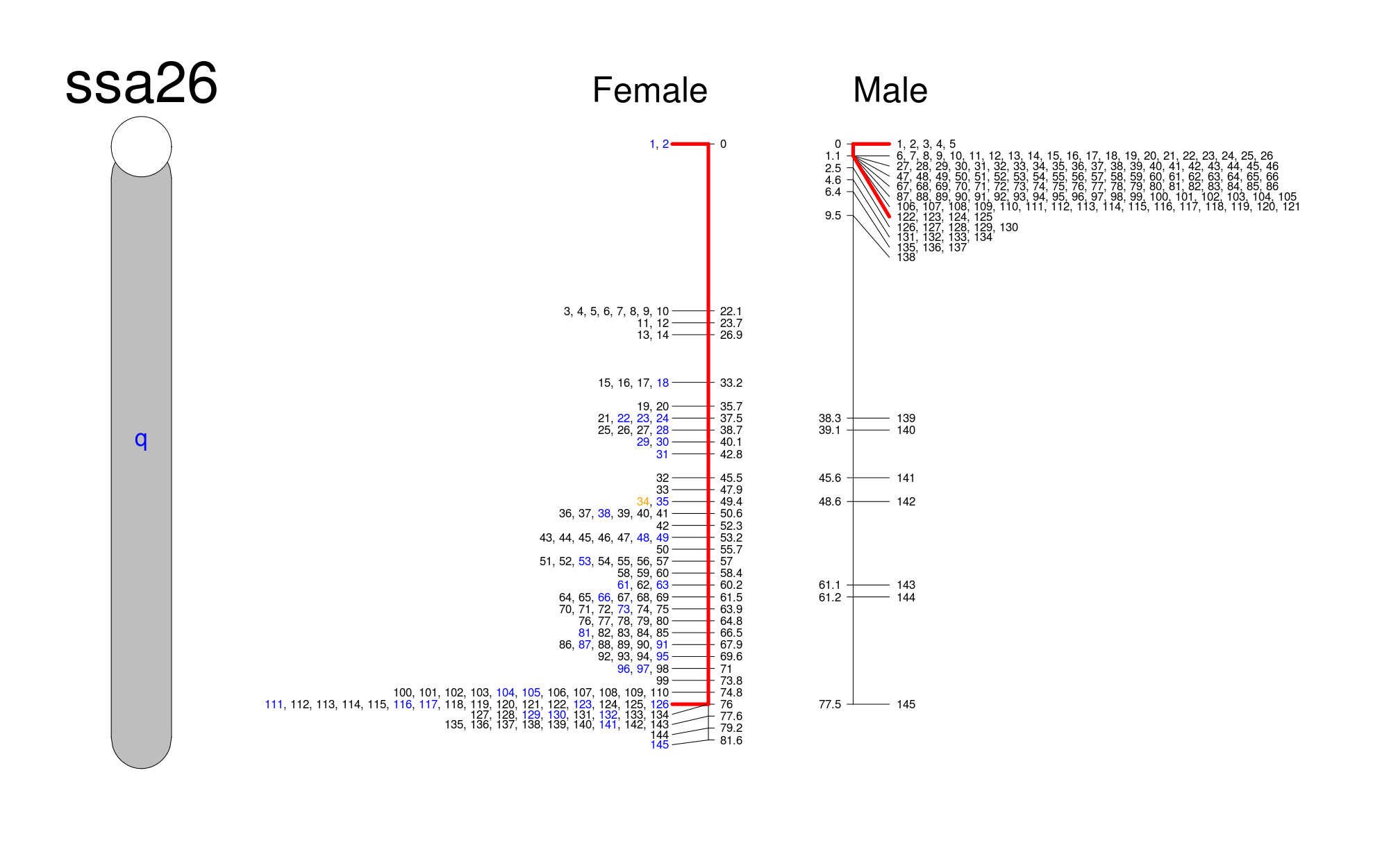

Supplement: Additional file 2 — Graphical visualization of linkage maps. The sections of the large acrocentric chromosomes proximal and distal to the central block of repetitive DNA are labeled qa and qb, respectively. The largest acrocentric chromosome pair has two blocks of repetitive DNA dividing the arm into three parts: 9qa, 9qb and 9qc. [file 1471-2164-12-615-S2.ZIP › ssa26.png]

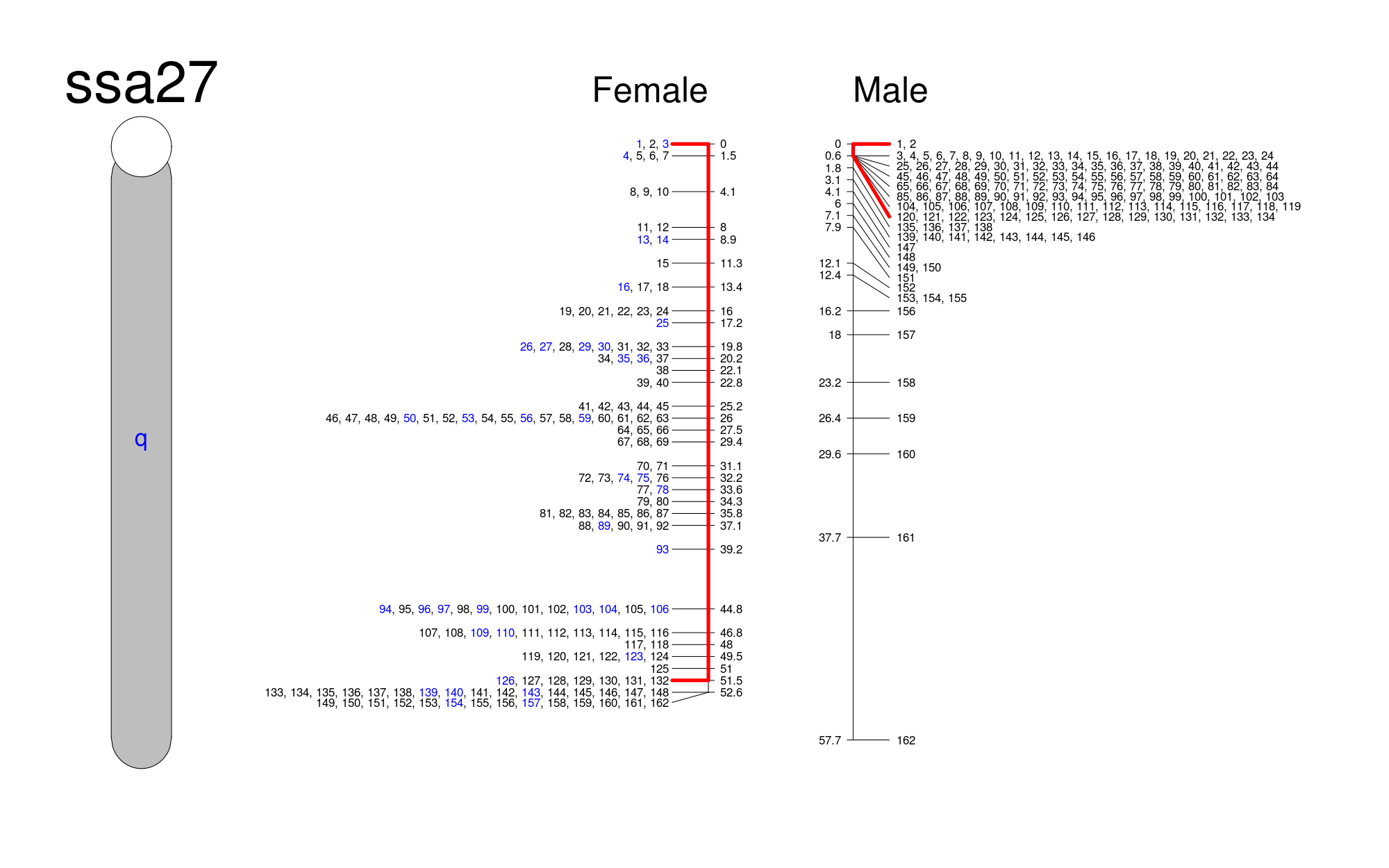

Supplement: Additional file 2 — Graphical visualization of linkage maps. The sections of the large acrocentric chromosomes proximal and distal to the central block of repetitive DNA are labeled qa and qb, respectively. The largest acrocentric chromosome pair has two blocks of repetitive DNA dividing the arm into three parts: 9qa, 9qb and 9qc. [file 1471-2164-12-615-S2.ZIP › ssa27.png]

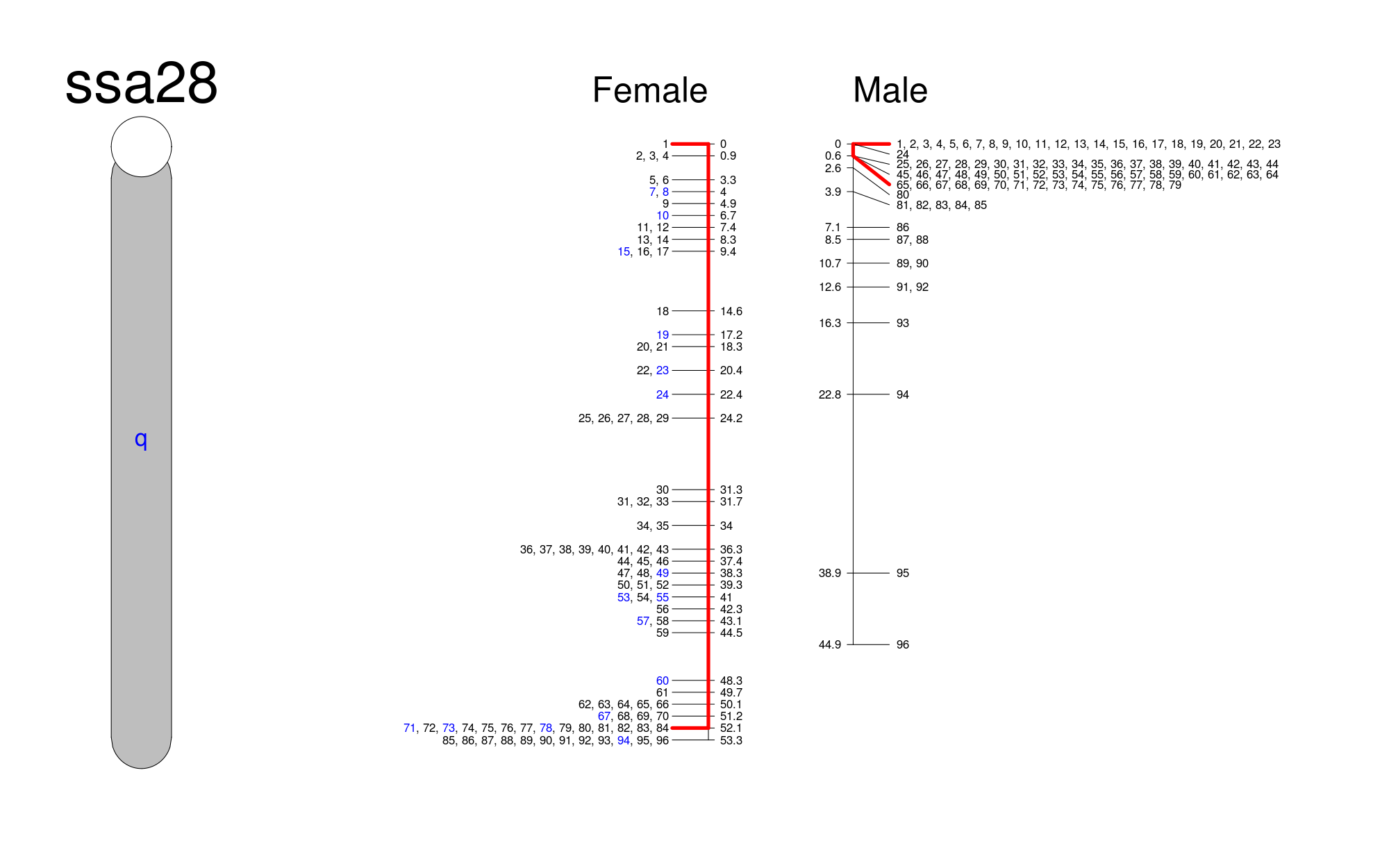

Supplement: Additional file 2 — Graphical visualization of linkage maps. The sections of the large acrocentric chromosomes proximal and distal to the central block of repetitive DNA are labeled qa and qb, respectively. The largest acrocentric chromosome pair has two blocks of repetitive DNA dividing the arm into three parts: 9qa, 9qb and 9qc. [file 1471-2164-12-615-S2.ZIP › ssa28.png]

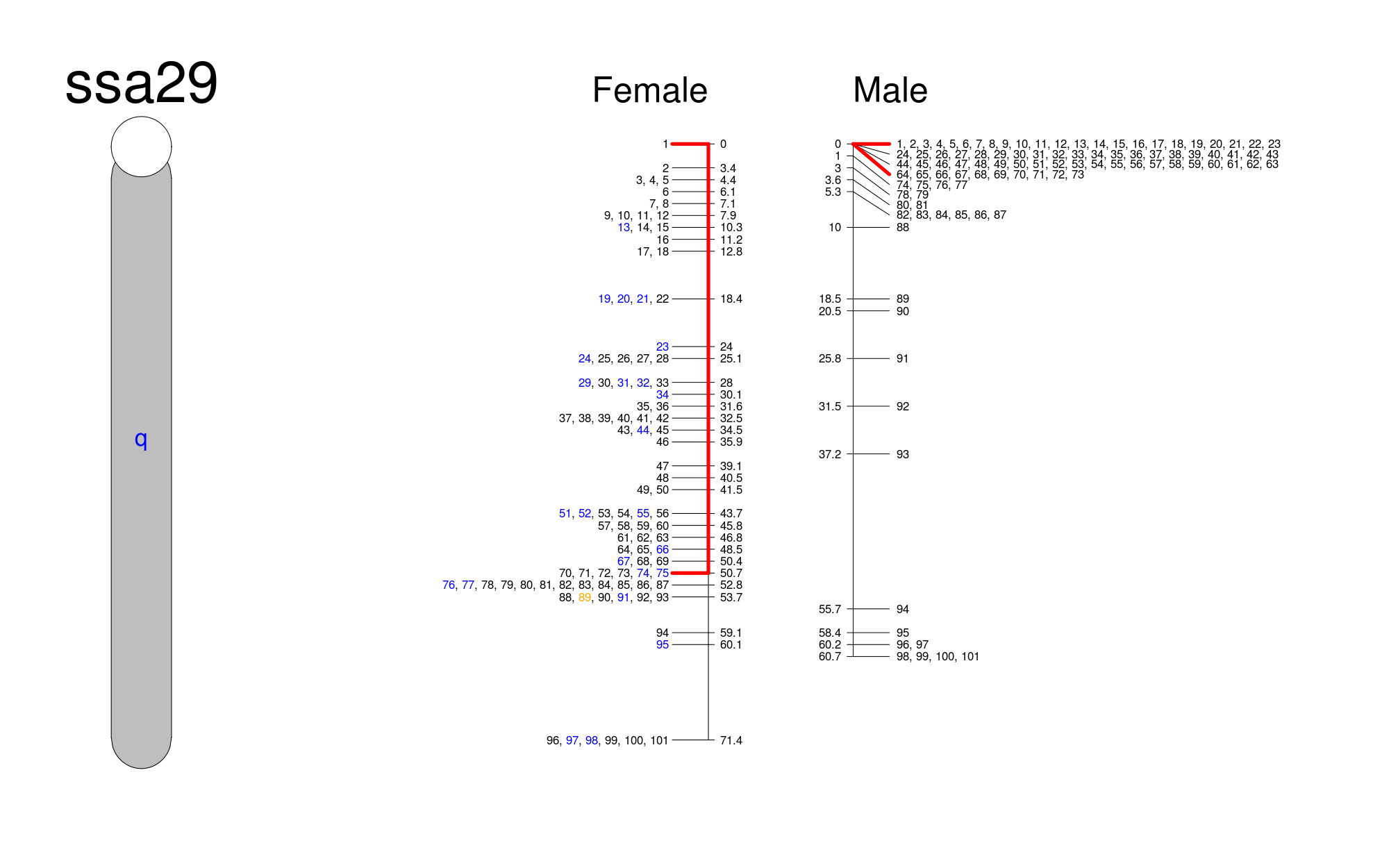

Supplement: Additional file 2 — Graphical visualization of linkage maps. The sections of the large acrocentric chromosomes proximal and distal to the central block of repetitive DNA are labeled qa and qb, respectively. The largest acrocentric chromosome pair has two blocks of repetitive DNA dividing the arm into three parts: 9qa, 9qb and 9qc. [file 1471-2164-12-615-S2.ZIP › ssa29.png]
